# Influences of 5‐Hydroxytryptamine Receptor Agonists on Spermatozoal Hyperactivation and In Vitro Fertilization Success in Mice

**DOI:** 10.1002/rmb2.70092

**Published:** 2026-09-16

**Authors:** Gaku Nakamura, Masakatsu Fujinoki, Takao Kamai

**Affiliations:** ^1^ Department of Urology Dokkyo Medical University Mibu Tochigi Japan; ^2^ Laboratory for Reproductive Medicine, Research Center for Advanced Medical Science Dokkyo Medical University Mibu Tochigi Japan

**Keywords:** 5‐hydroxytryptamine, hyperactivation, in vitro fertilization, mouse, spermatozoa

## Abstract

**Purpose:**

In mice, 5‐hydroxytryptamine (5‐HT) enhances hyperactivation through 5‐HT_2_, 5‐HT_3_, 5‐HT_4_, and 5‐HT_7_ receptors and increases in vitro fertilization (IVF) success through the 5‐HT_4_ receptor. In the present study, we examined the effects of 5‐HT receptor agonists on motility, hyperactivation, motility kinematics, and IVF success in mice.

**Methods:**

Spermatozoa were incubated for 4 h at 37°C under 5% CO_2_ in modified Tyrode's albumin lactate pyruvate medium to induce hyperactivation. Motility and hyperactivation were analyzed using a video microscope. Motility kinematics were evaluated using the Sperm Motility Analysis System. IVF was performed by co‐incubation of spermatozoa and oocytes for 0.5 and 5 h.

**Results:**

5‐HT receptors were detected. 5‐HT_2_, 5‐HT_2A_, 5‐HT_2B_, and 5‐HT_4_ receptor agonists significantly enhanced hyperactivation and increased IVF success. 5‐HT_3_ and 5‐HT_7_ receptor agonists slightly enhanced hyperactivation but did not affect IVF success. These agonists did not affect motility. During hyperactivation, the velocities of motility kinematics decreased. 5‐HT_2_, 5‐HT_2A_, 5‐HT_2B_, and 5‐HT_4_ receptor agonists accelerated the decrease in velocities.

**Conclusion:**

These results suggest that 5‐HT_2_ and 5‐HT_4_ receptors participate in the enhancement of hyperactivation and the increase in IVF success in mice. The influence of the 5‐HT_4_ receptor agonist is stronger than that of the 5‐HT_2_ receptor agonists.

## 1 Introduction

5‐hydroxytryptamine (5‐HT) is secreted from neurons and regulates mood. 5‐HT is synthesized from tryptophan via two‐phase reactions that are associated with tryptophan hydroxylase and aromatic L‐amino acid decarboxylase. 5‐HT influences various cellular functions through 5‐HT receptors [1, 2]. 5‐HT receptors consist of seven types (5‐HT_1–7_) [1, 2]. Moreover, the 5‐HT_1_ receptor has five subtypes (5‐HT_1A_, 5‐HT_1B_, 5‐HT_1D_, 5‐HT_1E_, and 5‐HT_1F_). The 5‐HT_2_ receptor has three subtypes (5‐HT_2A–2C_). The 5‐HT_1_ and 5‐HT_5_ receptors suppress the activation of transmembrane adenylate cyclase (tmAC), whereas 5‐HT_4_, 5‐HT_6_, and 5‐HT_7_ stimulate tmAC. Activation of tmAC increases cAMP concentration, inducing the activation of protein kinase A (PKA). The 5‐HT_2_ receptors stimulate phospholipase C and increase intracellular Ca^2+^ concentration. The 5‐HT_3_ receptor is a ligand‐gated cation channel that induces depolarization.

5‐HT and 5‐HT receptors are found in reproductive organs [2, 3, 4, 5, 6, 7]. In rats, it was reported that the 5‐HT concentration in the oviduct was between 2.06 and 3.34 μg/g fresh tissue [8]. In humans, it was reported that 5‐HT concentrations in preovulatory follicles and cystically degenerated follicles were 14.3 ± 8.9 μg/100 mL and 12.2 ± 6.2 μg/100 mL, respectively [9]. In the female reproductive organ, 5‐HT is released from cumulus cells and participates in steroidogenesis and oocyte maturation [2]. 5‐HT induces the release of progesterone from human granulosa cells [10]. In male reproductive organs, 5‐HT is released from Leydig cells, binds to the 5‐HT_2_ receptor, and reduces androgen secretion [11, 12, 13]. Moreover, 5‐HT regulates seasonal breeding through gonadotropin‐induced cAMP signaling in hamsters [12]. Additionally, 5‐HT, 5‐HT receptors, tryptophan hydroxylase, and aromatic L‐amino acid decarboxylase are found in hamster, human, and rat spermatozoa [5, 7, 14]. Moreover, recent studies [5, 14] showed that hamster and human spermatozoa synthesize 5‐HT from tryptophan and secrete 5‐HT.

In hamster spermatozoa, 5‐HT induced the acrosome reaction (AR) and enhanced hyperactivation through the 5‐HT_2_ and the 5‐HT_4_ receptor [3, 4]. Several tens of μM 5‐HT induced AR through the 5‐HT_4_ receptor [3]. Conversely, 5‐HT enhanced spermatozoal hyperactivation in a dose‐dependent manner [4]. 5‐HT at fM to pM enhanced hyperactivation through the 5‐HT_2_ receptor, whereas 5‐HT at nM to μM enhanced hyperactivation through the 5‐HT_4_ receptor. Moreover, hamster spermatozoa synthesize 5‐HT from tryptophan [14]. Sperm‐synthesized 5‐HT enhanced hyperactivation through the 5‐HT_4_ receptor [14]. In mouse spermatozoa, 5‐HT enhanced hyperactivation through 5‐HT_2_, 5‐HT_3_, 5‐HT_4_, and 5‐HT_7_ receptors [6]. In rat spermatozoa, 5‐HT enhanced hyperactivation through the 5‐HT_4_ receptor only [7]. In human spermatozoa, 5‐HT increased velocity associated with progressive motility [5].

AR and hyperactivation are spermatozoal events associated with capacitation in mammals [15, 16, 17]. After ejaculation, spermatozoa are not able to fertilize an oocyte, whereas they acquire fertilizing ability in the oviduct. The process by which spermatozoa acquire fertilizing ability is called capacitation. AR is an exocytotic process that involves the release of acrosomal vesicles [15, 16]. Following AR, spermatozoa digest the oocyte envelopes and then bind to the oocyte. Hyperactivation is a specialized motility exhibited by capacitated spermatozoa [15, 17]. Before hyperactivation, spermatozoa exhibit symmetric flagellar movement, whereas they exhibit asymmetric flagellar movement after hyperactivation [15]. In addition, the flagellar movement of hyperactivated spermatozoa shows larger amplitude and low progressive motility. Hyperactivated spermatozoa swim through the viscous oviductal fluid and penetrate oocyte envelopes [15, 17]. Notably, it has been suggested that hyperactivation capacity is correlated with in vitro fertilization (IVF) success [18, 19, 20, 21]. In mice [6] and rats [7], 5‐HT enhanced hyperactivation and increased IVF success through the 5‐HT_4_ receptor.

Previously, we reported that 5‐HT enhanced mouse spermatozoal hyperactivation via 5‐HT_2_, 5‐HT_3_, 5‐HT_4_, and 5‐HT_7_ receptors [6]. In addition, 5‐HT increased mouse IVF success, and the increase in IVF success by 5‐HT was inhibited by an antagonist of the 5‐HT_4_ receptor [6]. In the present study, we examined the effects of 5‐HT receptor agonists on spermatozoal hyperactivation and IVF success. In addition, we examined which 5‐HT_2_ receptor subtype affected spermatozoal hyperactivation and IVF.

## 2 Materials and Methods

### 2.1 Chemicals

Bovine serum albumin (BSA), 5‐HT, α‐methylserotonin maleate (MS), 1‐(3‐chlorophenyl) biguanide hydrochloride (mCPBG), 5‐methoxytryptamine (MT), LP12 hydrochloride hydrate (LP12), cyproheptadine hydrochloride sesquihydrate (Cypro), dolasetron mesylate hydrate (DS), GR113808 (GR), and SB‐258719 (SB) were purchased from Merck KGaA (Darmstadt, Germany). TCB2, BW723C86 (BW), MK212, LY272015 (LY), and MDL11939 (MDL) were purchased from TOCRIS Bioscience (Bristol, UK). Pregnant mare serum gonadotropin (PMSG) (Serotropin) and human chorionic gonadotropin (hCG) (Gonatropin) were purchased from ASKA Animal Health Co. Ltd. (Tokyo, Japan). Anti 5‐HT_2A_ receptor antibody (SR‐2A(A‐4): sc‐166775), anti 5‐HT_2B_ receptor antibody (SR‐2B(C‐6): sc‐376878), anti 5‐HT_2C_ receptor antibody (SR‐2C(D‐12): sc‐17797), anti 5‐HT_3A_ receptor antibody (SR‐3A(A‐9): sc‐390168), anti 5‐HT_3B_ receptor antibody (SR‐3B(H‐9): sc‐390642), anti 5‐HT_4_ receptor antibody (SR‐4(G‐3): sc‐376158) were purchased from Santa Cruz Biotechnology Inc. (Texas, USA). Anti 5‐HT_7_ receptor antibody (EPR6271: ab128892) was purchased from Abcam Ltd. (Cambridge, UK). Polyvinylidene difluoride membranes were purchased from Millipore (Bedford, MA, USA). The molecular weight marker set was purchased from Bio‐Rad Laboratories Inc. (Hercules, CA, USA). EzWestLumi Plus was purchased from the ATTO Corporation (Tokyo, Japan). Other reagent‐grade chemicals were purchased from FUJIFILM Wako Pure Chemical Corporation (Osaka, Japan).

### 2.2 Animals

ICR mice were purchased from Japan SLC Inc. (Hamamatsu, Shizuoka, Japan) and were propagated at the Research Center for Laboratory Animals, Dokkyo Medical University. Mice were housed in a light‐ and temperature‐controlled facility (12 h on/12 h off and 25°C ± 2°C, respectively) with food and water available *ad libitum*. All experiments were approved by the Animal Care and Use Committee of the University (experimental permission number: 0107) and were performed according to the University guidelines for animal experimentation.

### 2.3 Preparation of Hyperactivated Spermatozoa

Hyperactivated spermatozoa were prepared as previously described [6]. The capacitation medium was modified Tyrode's albumin lactate pyruvate (mTALP) medium [22], which contains 101.02 mm NaCl, 2.68 mm KCl, 2 mm CaCl_2_·2H_2_O, 1.5 mm MgCl_2_·6H_2_O, 360 μm NaH_2_PO_4_·2H_2_O, 35.70 mm NaHCO_3_, 4.5 mm D‐glucose, 90 μm sodium pyruvate, 9 mm sodium lactate, 500 μm hypotaurine, 50 μm (±)‐epinephrine hydrochloride, 200 μm sodium taurocholate, 5.26 μm sodium metabisulfite, 0.05% (w/v) streptomycin sulfate, 0.05% (w/v) potassium penicillin G, and 15 mg/mL BSA (pH 7.4 at 37°C under 5% (v/v) CO_2_ in air). To harvest spermatozoa, the caudal epididymis of a mature mouse (10–24 weeks old) was punctured with a 23 G (0.6 mm) needle (Terumo Corporation, Tokyo, Japan), and a drop of spermatozoa was obtained. This drop (approximately 3 μL) of caudal epididymal spermatozoa was placed in a culture dish (35 mm diameter; Iwaki, Asahi Glass Co. Ltd., Tokyo, Japan), and 3 mL of mTALP medium was added. The spermatozoa were incubated for 5 min at 37°C for activation. The supernatant, which contained motile spermatozoa, was incubated for 4 h at 37°C and 5% CO_2_ to induce hyperactivation.

### 2.4 Treatment of 5‐HT, 5‐HT Receptor Agonists, and 5‐HT Receptor Antagonists to Spermatozoa

5‐HT, 5‐HT receptor agonists, and 5‐HT receptor antagonists were applied to spermatozoa as described below. After spermatozoa were activated, they were placed in a new dish containing vehicle or 5‐HT receptor antagonists. After incubation for 5 min, the supernatant was transferred to a new dish containing vehicle, 5‐HT, or 5‐HT receptor agonists, and the spermatozoa were incubated for 4 h at 37°C and 5% CO_2_ to induce hyperactivation. Stock solutions of 5‐HT (1 mM), MS (100 pM), mCPBG (100 mM), LP12 (130 nM), TCB2 (750 nM), MK212 (300 nM), and DS (3.8 μM) were dissolved in pure water. MT (10 nM) and Cypro (1 mM) were dissolved in ethanol. BW (2 mM), GR (1 mM), LY (750 nM), MDL (540 nM), and SB (10 mM) were dissolved in dimethyl sulfoxide. In all experiments, the maximum concentration of the vehicle was 0.2% (v/v).

### 2.5 Measurements of Motility and Hyperactivation

Motile and hyperactivated spermatozoa were analyzed as previously described [6]. Motile spermatozoa were recorded to a Blu‐ray disc recorder (AQUOS ZB‐C20ET1; SHARP, Osaka, Japan) using a CCD camera (Progressive 3CCD; Sony, Tokyo, Japan) attached to a microscope (IX70; Olympus, Tokyo, Japan) under phase‐contrast illumination in a small CO_2_ incubator (MI‐IBC; Olympus). Spermatozoa were recorded for 1 min at 37°C after incubation for 0, 0.5, 1, 1.5, 2, 2.5, 3, and 4 h, respectively. The movies were replayed to manually count the total spermatozoa, motile spermatozoa, and hyperactivated spermatozoa from two different fields at one observation point. The experiments were repeated four times using four mice each. Visual analyses were performed blindly. Motile spermatozoa that exhibited a whiplash‐like trajectory were considered hyperactivated [6, 15, 23]. The percentages of motility and hyperactivation were calculated as the number of motile spermatozoa/total spermatozoa × 100 and the number of hyperactivated spermatozoa/total spermatozoa × 100, respectively. The experiment was repeated when the percentage of motile spermatozoa was ≤ 80%.

### 2.6 Motility Kinematic Analysis

Motility kinematic analyses were evaluated using the sperm motility analysis system (SMAS) for animals (Ver. 3.18) with the loaded parameter file mouse_BM10 × _640 nm_Bright59_150fps‐shutter200.ini (Ditect Co. Ltd., Tokyo, Japan), as previously described [6]. Spermatozoa were hyperactivated according to the methodology described in the section “Preparation of Hyperactivated Spermatozoa.” The suspension containing motile spermatozoa (20 μL) was transferred to an observation chamber (0.1 mm deep, 18 mm wide, and 18 mm long), which was made using vinyl tape and a cover glass. After incubation for 0, 0.5, 1, 1.5, 2, 2.5, 3, and 4 h, respectively, spermatozoal movement was recorded at 37°C for 1 s using the SMAS with a microscope (ECLIPSE E200; Nikon Corp., Tokyo, Japan) equipped with phase‐contrast illumination, a 650 nm band‐pass filter, and a warm plate (MP10DM; Kitazato Corp., Shizuoka, Japan). The SMAS automatically calculated straight‐line velocity (VSL; μm/s), curvilinear velocity (VCL; μm/s), average‐path velocity (VAP; μm/s), linearity (LIN), straightness (STR), amplitude of lateral head displacement (ALH; μm), and beat‐cross frequency (BCF; Hz). The wobble coefficient (WOB), defined as VAP/VCL [24], was calculated manually. The SMAS analysis was repeated six times using one mouse each time. In each experiment, ≥ 300 spermatozoa were analyzed.

### 2.7 Preparation of the Spermatozoal Protein Extract

Spermatozoal proteins were extracted as previously described [25]. Spermatozoa were homogenized at 10 mg spermatozoa (wet weight)/ml in a urea solution containing 7 M urea and 10% (v/v) 2‐mercaptoethanol, incubated on ice for 10 min, and centrifuged at 15,000 × g for 10 min at 4°C. The supernatant was used as the urea extract. The precipitate was re‐homogenized in a urea–thiourea solution at the same volume as the urea solution. The urea–thiourea solution contained 5 M urea, 1 M thiourea, 10% (v/v) 2‐mercaptoethanol, and 2% (v/v) NP‐40. The homogenate was incubated on ice for 10 min and centrifuged at 15,000 × g for 10 min at 4°C. The resulting supernatant was used as the urea–thiourea extract.

### 2.8 Sodium Dodecyl Sulfate‐Polyacrylamide Gel Electrophoresis (SDS‐PAGE)

SDS‐PAGE was conducted as described by Laemmli [26] using a separating gel of 10% (w/v) polyacrylamide containing 0.1% (w/v) SDS. Following Coomassie brilliant blue staining, the gel was scanned using a densitometer (GS‐800; Bio‐Rad Laboratories).

### 2.9 Western Blotting

Western blotting was performed as previously described [27]. After SDS‐PAGE, the proteins were transferred onto polyvinylidene fluoride membranes. After washing with Tris‐buffered saline (TBS) containing 0.15 m NaCl and 20 mm Tris–HCl (pH 7.5), the membranes were incubated with BSA‐TBS containing 5% (w/v) BSA and TBS for 1 h at 25°C. Following three washes with TBS, the membranes were incubated with primary antibodies for 1 h at 25°C (anti‐5‐HT_2A_ receptor antibody, anti‐5‐HT_2B_ receptor antibody, anti‐5‐HT_2C_ receptor antibody, anti‐5‐HT_3A_ receptor antibody, anti‐5‐HT_3B_ receptor antibody, and anti‐5‐HT_4_ receptor antibody, diluted 200‐fold in BSA‐TBS and anti‐5‐HT_7_ receptor antibody diluted 500‐fold in BSA‐TBS). After washing thrice with TBS, the membranes were incubated with peroxidase‐conjugated secondary antibodies (1:5000 dilution with BSA‐TBS). The membrane was washed three times with 0.05% (w/v) Tween‐20 containing TBS. Chemiluminescence was detected using EzWestLumi plus, and antibody reactivity was analyzed using Ez‐Capture MG (ATTO).

### 2.10 IVF

Mouse IVF was performed as described previously [6, 28]. Superovulation treatment was performed as follows: female mice (8–12 weeks old) were intraperitoneally administered 10 U of PMSG. Approximately 48 h after PMSG injection, female mice were intraperitoneally administered 30 U of hCG. IVF was performed 18 h after the hCG injection. One hour before cumulus–oocyte complex (COC) collection, one drop (approximately 3 μL) of the dense sperm mass, obtained from the caudal epididymides of male mice (10–20 weeks old), was mixed with 300 μL drops of mTALP medium with or without vehicle, 5‐HT receptor agonists, or 5‐HT receptor antagonists. Spermatozoa (2 × 10^7^ cells/ml) were incubated for 1 h at 37°C in an atmosphere of 5% CO_2_. Eighteen hours after hCG injection, COCs were collected from PMSG/hCG‐treated female mice. COCs were dissected from the ampullae of both oviducts and incubated in 300 μL drops of mTALP medium with or without vehicle, agonists, or antagonists. After COC collection and 1 h of spermatozoal incubation, 10 μL of the suspension containing the pre‐incubated spermatozoa was added to the medium containing COCs to start the insemination process. The final spermatozoal concentration was 6.7 × 10^5^ cells/ml. Spermatozoa and COCs were incubated together for 0.5 or 5 h at 37°C in an atmosphere of 5% CO_2_. After incubation, oocytes or COCs were collected and washed with mTALP medium with or without vehicle, agonists, or antagonists to remove spermatozoa and cumulus cells, and insemination was stopped. After washing, the oocytes or COCs were incubated again for 18–24 h, and the cells were observed under a stereoscopic microscope (Stemi 2000‐C; Zeiss, Oberkochen, Germany). Eggs and two‐cell embryos were counted manually. The percentage of two‐cell embryos was calculated as the number of two‐cell embryos/the number of eggs × 100. Experiments were performed four times using one female and one male mouse each time.

### 2.11 Statistical Analyses

**FIGURE 1:**
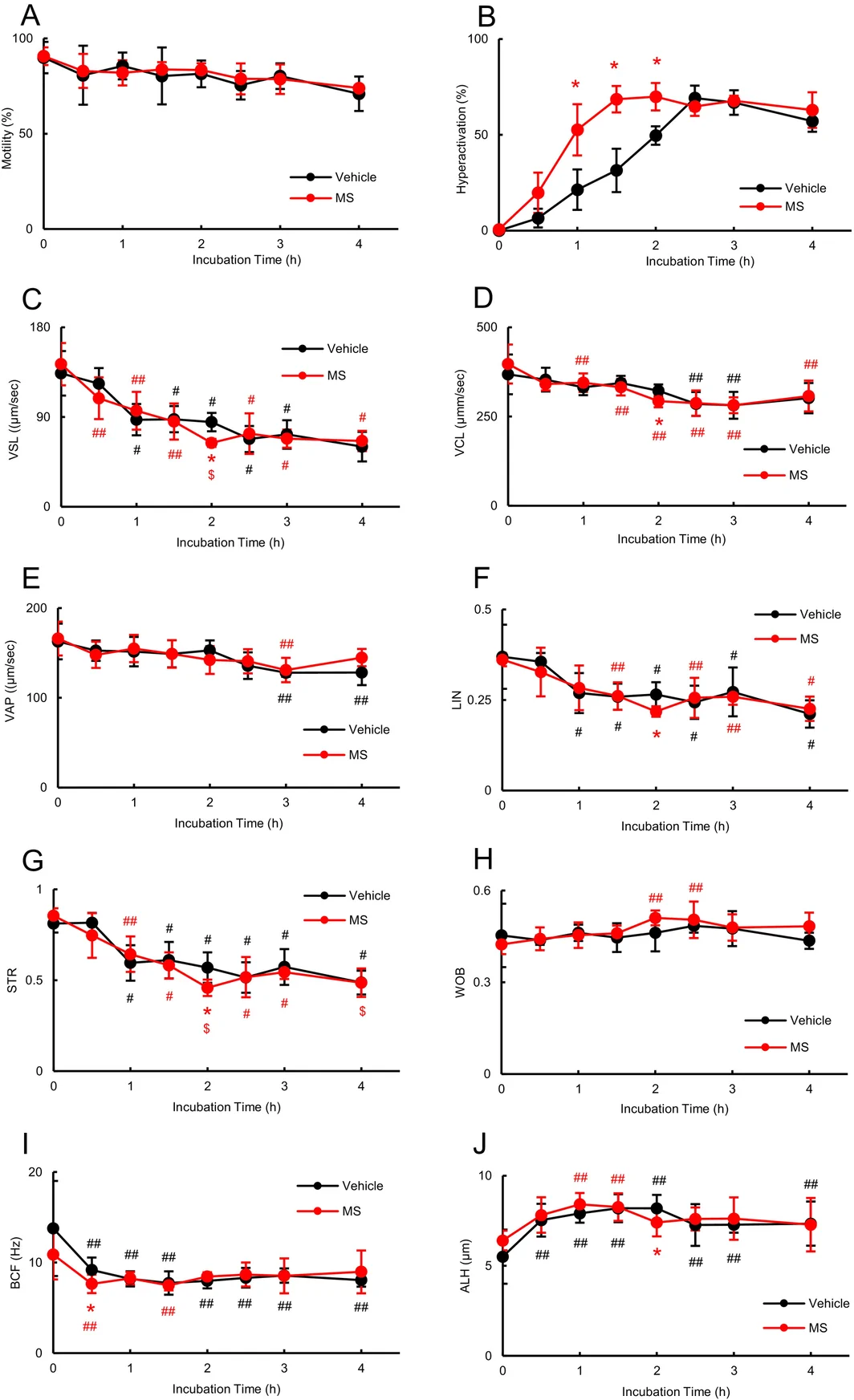
Effects of α‐methylserotonin maleate (MS) on spermatozoal motility, hyperactivation, and motility kinematics. Percentages of motility (A) and hyperactivation (B) are shown when spermatozoa were cultured for 4 h in the presence or absence of MS. Experiments were repeated four times using spermatozoa from four different male mice. As for motility kinematics, straight‐line velocity (VSL) (C), curvilinear velocity (VCL) (D), average‐path velocity (VAP) (E), linearity (LIN) (F), straightness (STR) (G), wobble coefficient (WOB) (H), beat‐cross frequency (BCF) (I), and amplitude of lateral head displacement (ALH) (J) are shown when spermatozoa were cultured for 4 h in the presence or absence of MS. Motility kinematic analyses were repeated six times using spermatozoa from six different male mice. Data represent the mean ± standard deviation. (A–J): (Vehicle) medium containing 0.1% pure water as the vehicle; (MS) medium containing 100 fM MS and vehicle. *Significant differences compared with Vehicle in the same time (*p* < 0.01). ^#^Significant differences compared with 0 and 0.5 h in the same condition (*p* < 0.01). ^##^Significant differences compared with 0 h in the same condition (*p* < 0.01). ^$^Significant differences compared with 0, 0.5, and 1 h in the same condition (*p* < 0.01).

**FIGURE 2:**
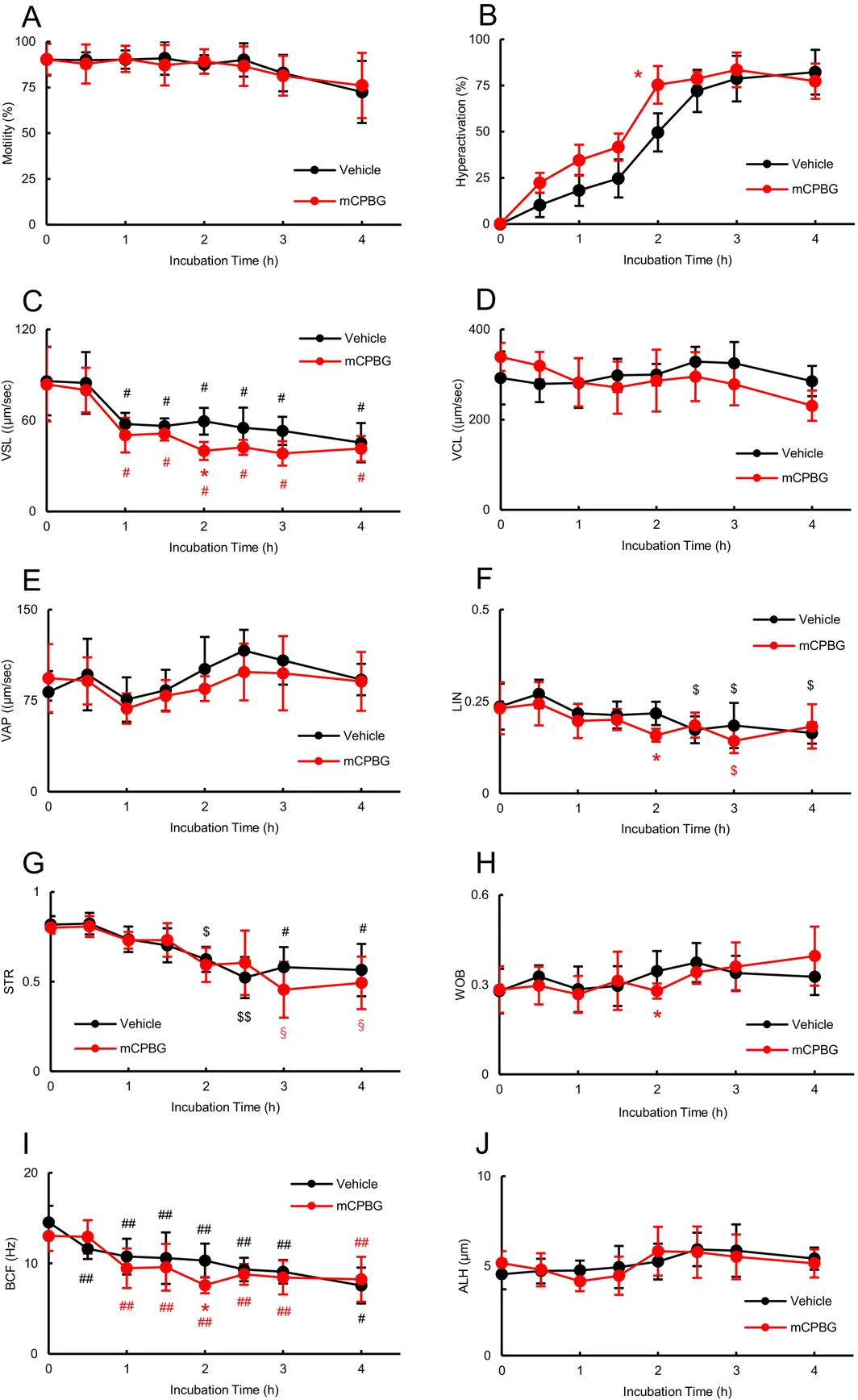
Effects of 1‐(3‐chlorophenyl) biguanide hydrochloride (mCPBG) on spermatozoal motility, hyperactivation, and motility kinematics. Percentages of motility (A) and hyperactivation (B) are shown when spermatozoa were cultured for 4 h in the presence or absence of mCPBG. Experiments were repeated four times using spermatozoa from four different male mice. As for motility kinematics, straight‐line velocity (VSL) (C), curvilinear velocity (VCL) (D), average‐path velocity (VAP) (E), linearity (LIN) (F), straightness (STR) (G), wobble coefficient (WOB) (H), beat‐cross frequency (BCF) (I), and amplitude of lateral head displacement (ALH) (J) are shown when spermatozoa were cultured for 4 h in the presence or absence of mCPBG. Motility kinematic analyses were repeated six times using spermatozoa from six different male mice. (A–J): (Vehicle) medium containing 0.1% pure water as the vehicle; (mCPBG) medium containing 100 μM mCPBG and vehicle. *Significant differences compared with Vehicle in the same time (*p* < 0.01). ^#^Significant differences compared with 0 and 0.5 h in the same condition (*p* < 0.01). ^##^Significant differences compared with 0 h in the same condition (*p* < 0.01). ^$^Significant differences compared with 0.5 h in the same condition (*p* < 0.01). ^$$^Significant differences compared with 0, 0.5, and 1 h in the same condition (*p* < 0.01). ^§^Significant differences compared with 0, 0.5, 1, and 1.5 h in the same condition (*p* < 0.01).

**FIGURE 3:**
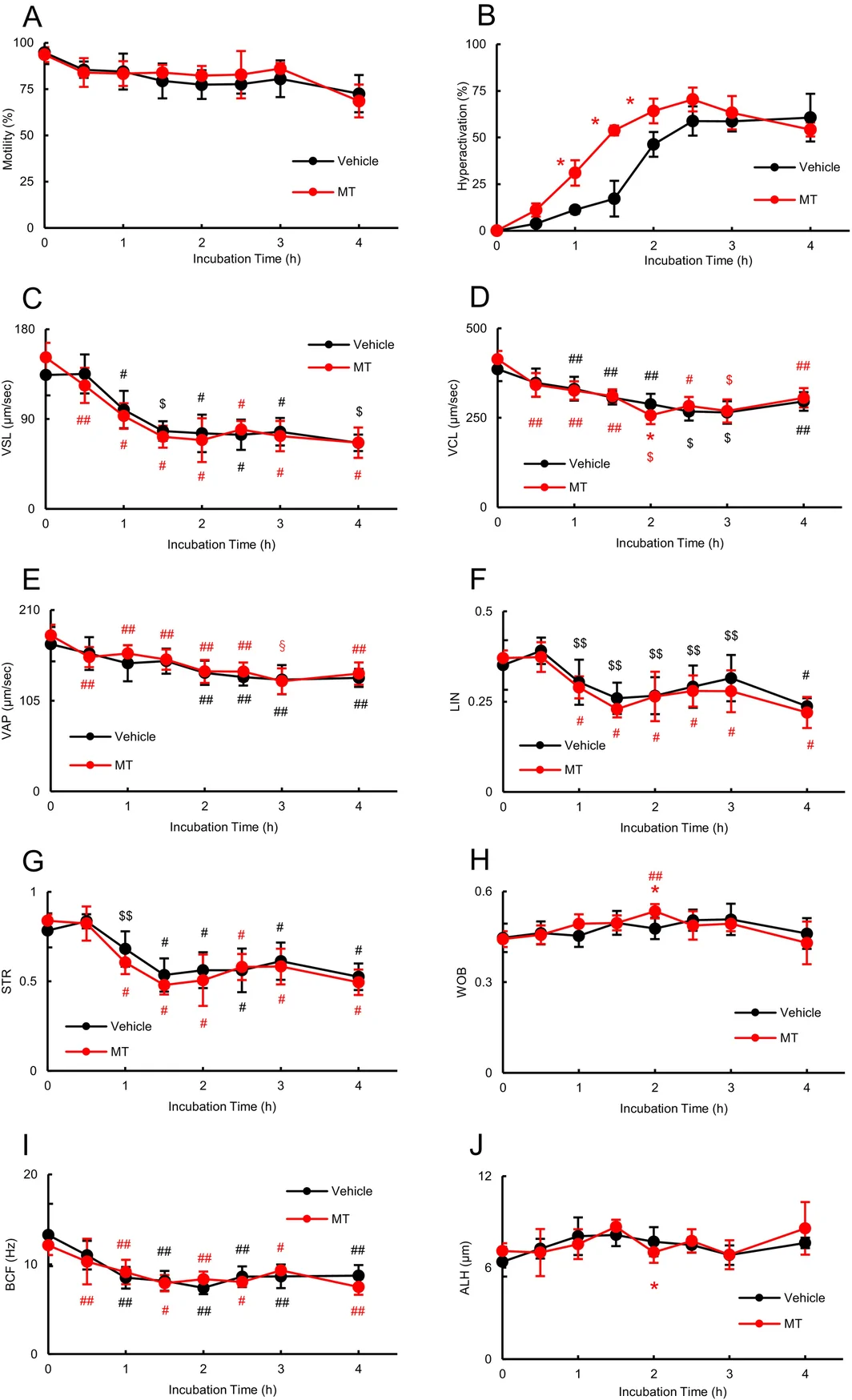
Effects of 5‐methoxytryptamine (MT) on spermatozoal motility, hyperactivation, and motility kinematics. Percentages of motility (A) and hyperactivation (B) are shown when spermatozoa were cultured for 4 h in the presence or absence of MT. Experiments were repeated four times using spermatozoa from four different male mice. As for motility kinematics, straight‐line velocity (VSL) (A), curvilinear velocity (VCL) (B), average‐path velocity (VAP) (C), linearity (LIN) (D), straightness (STR) (E), wobble coefficient (WOB) (F), beat‐cross frequency (BCF) (G), and amplitude of lateral head displacement (ALH) (H) are shown when spermatozoa were cultured for 4 h in the presence or absence of MT. Motility kinematic analyses were repeated six times using spermatozoa from six different male mice. Data represent the mean ± standard deviation. (A–J): (Vehicle) medium containing 0.1% ethanol as the vehicle; (MT) medium containing 10 pM MT and vehicle. *Significant differences compared with Vehicle in the same time (*p* < 0.01). ^#^Significant differences compared with 0 and 0.5 h in the same condition (*p* < 0.01). ^##^Significant differences compared with 0 h in the same condition (*p* < 0.01). ^$^Significant differences compared with 0, 0.5, and 1 h in the same condition (*p* < 0.01). ^$$^Significant differences compared with 0.5 h in the same condition (*p* < 0.01). ^§^Significant differences compared with 0, 0.5, 1, and 1.5 h in the same condition (*p* < 0.01).

**FIGURE 4:**
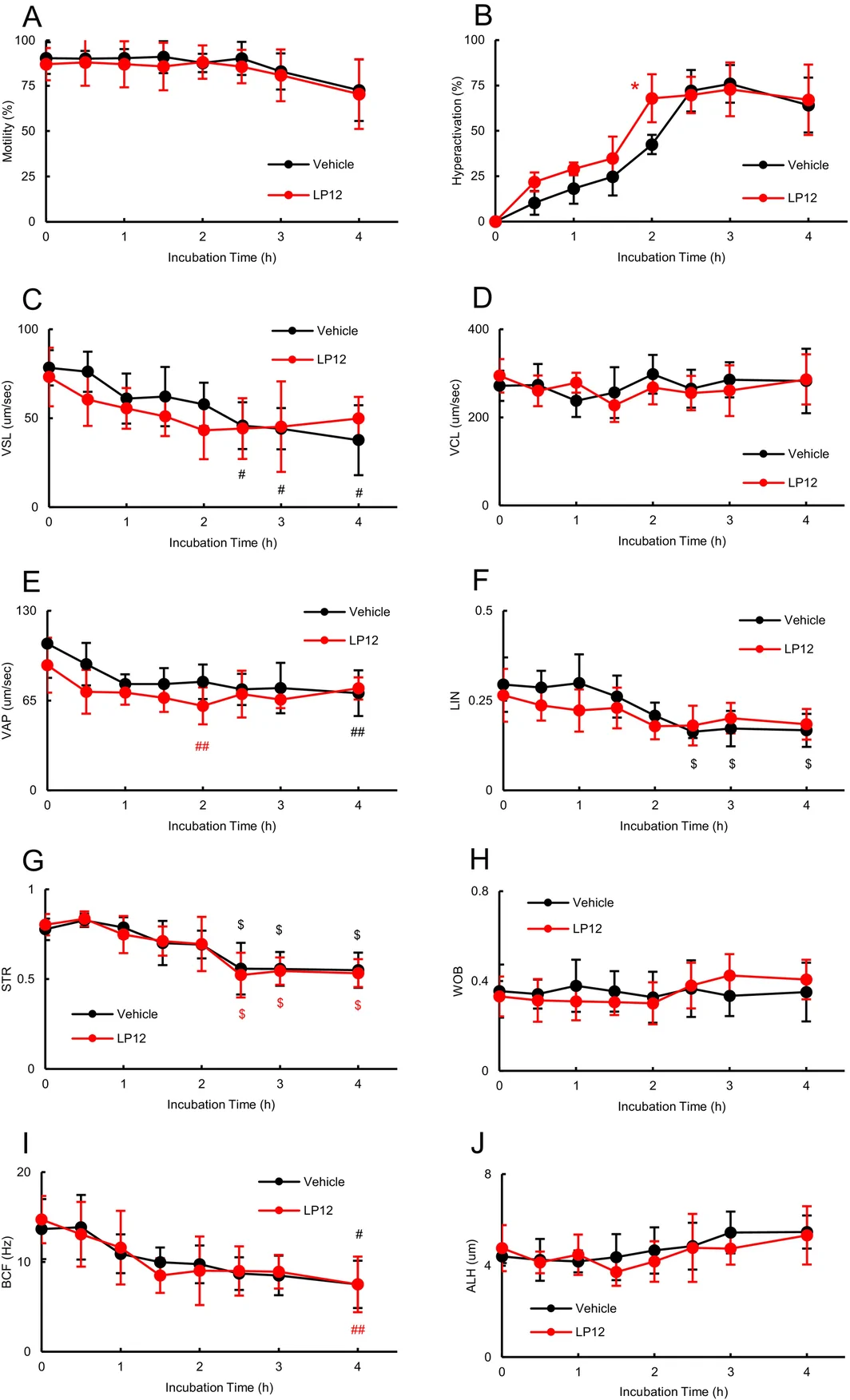
Effects of LP12 hydrochloride hydrate (LP12) on spermatozoal motility, hyperactivation, and motility kinematics. Percentages of motility (A) and hyperactivation (B) are shown when spermatozoa were cultured for 4 h in the presence or absence of LP12. Experiments were repeated four times using spermatozoa from four different male mice. As for motility kinematics, straight‐line velocity (VSL) (C), curvilinear velocity (VCL) (D), average‐path velocity (VAP) (E), linearity (LIN) (F), straightness (STR) (G), wobble coefficient (WOB) (H), beat‐cross frequency (BCF) (I), and amplitude of lateral head displacement (ALH) (J) are shown when spermatozoa were cultured for 4 h in the presence or absence of LP12. Motility kinematic analyses were repeated six times using spermatozoa from six different male mice. Data represent the mean ± standard deviation. (A–J): (Vehicle) medium containing 0.1% pure water as the vehicle; (LP12) medium containing 130 pM LP12 and vehicle. *Significant differences compared with Vehicle in the same time (*p* < 0.01). ^#^Significant differences compared with 0 and 0.5 h in the same condition (*p* < 0.01). ^##^Significant differences compared with 0 h in the same condition (*p* < 0.01). ^$^Significant differences compared with 0, 0.5, and 1 h in the same condition (*p* < 0.01).

**FIGURE 5:**
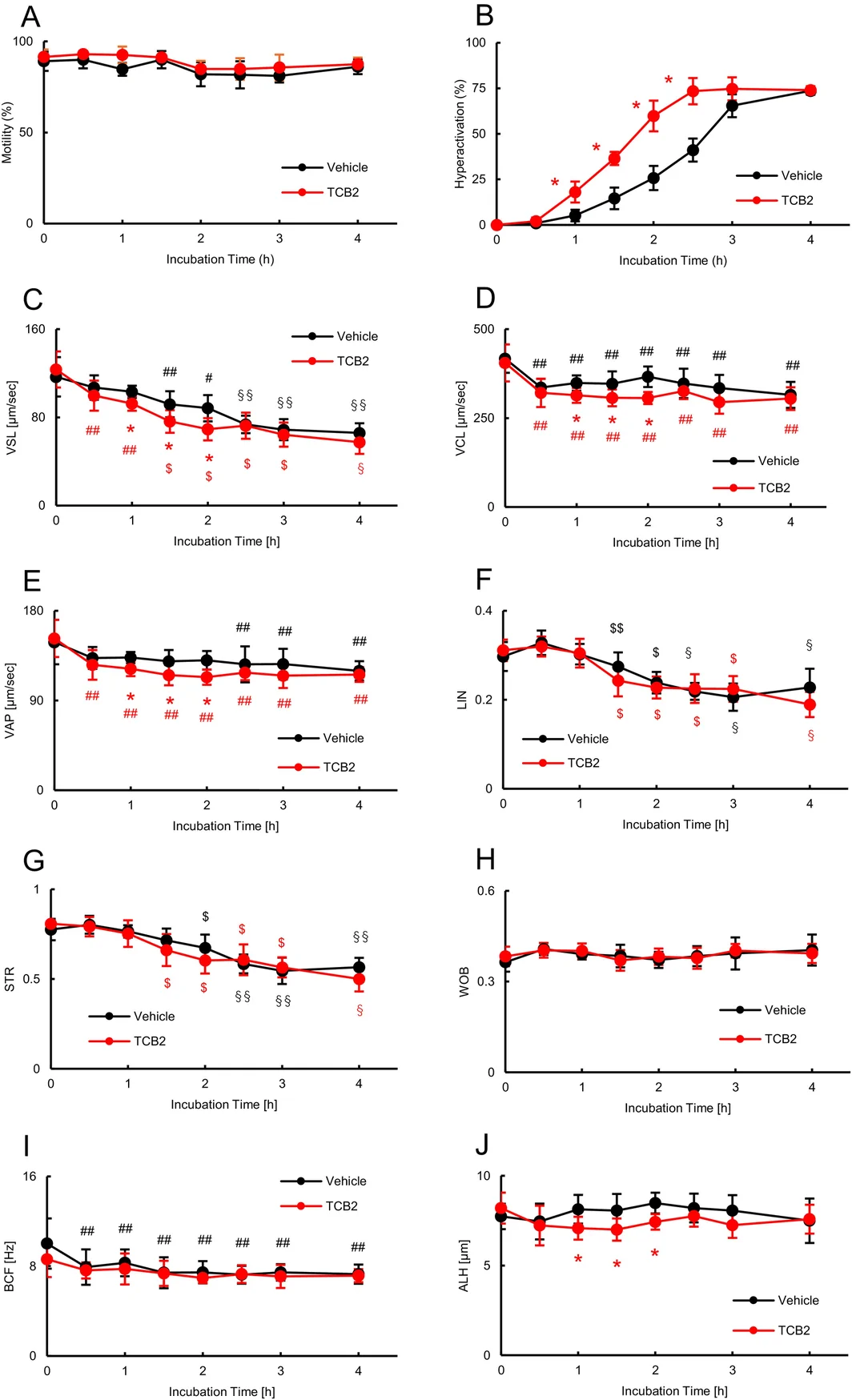
Effects of TCB2 on spermatozoal motility, hyperactivation, and motility kinematics. Percentages of motility (A) and hyperactivation (B) are shown when spermatozoa were cultured for 4 h in the presence or absence of TCB2. Experiments were repeated four times using spermatozoa from four different male mice. As for motility kinematics, straight‐line velocity (VSL) (C), curvilinear velocity (VCL) (D), average‐path velocity (VAP) (E), linearity (LIN) (F), straightness (STR) (G), wobble coefficient (WOB) (H), beat‐cross frequency (BCF) (I), and amplitude of lateral head displacement (ALH) (J) are shown when spermatozoa were cultured for 4 h in the presence or absence of TCB2. Motility kinematic analyses were repeated six times using spermatozoa from six different male mice. Data represent the mean ± standard deviation. (A–G): (Vehicle) medium containing 0.1% pure water as the vehicle; (TCB2) medium containing 750 pM TCB2 and vehicle. *Significant differences compared with Vehicle in the same time (*p* < 0.01). ^#^Significant differences compared with 0 and 0.5 h in the same condition (*p* < 0.01). ^##^Significant differences compared with 0 h in the same condition (*p* < 0.01). ^$^Significant differences compared with 0, 0.5, and 1 h in the same condition (*p* < 0.01). ^$$^Significant differences compared with 0.5 h in the same condition (*p* < 0.01). ^§^Significant differences compared with 0, 0.5, 1, and 1.5 h in the same condition (*p* < 0.01). ^§§^Significant differences compared with 0, 0.5, 1, 1.5, and 2 h in the same condition (*p* < 0.01).

**FIGURE 6:**
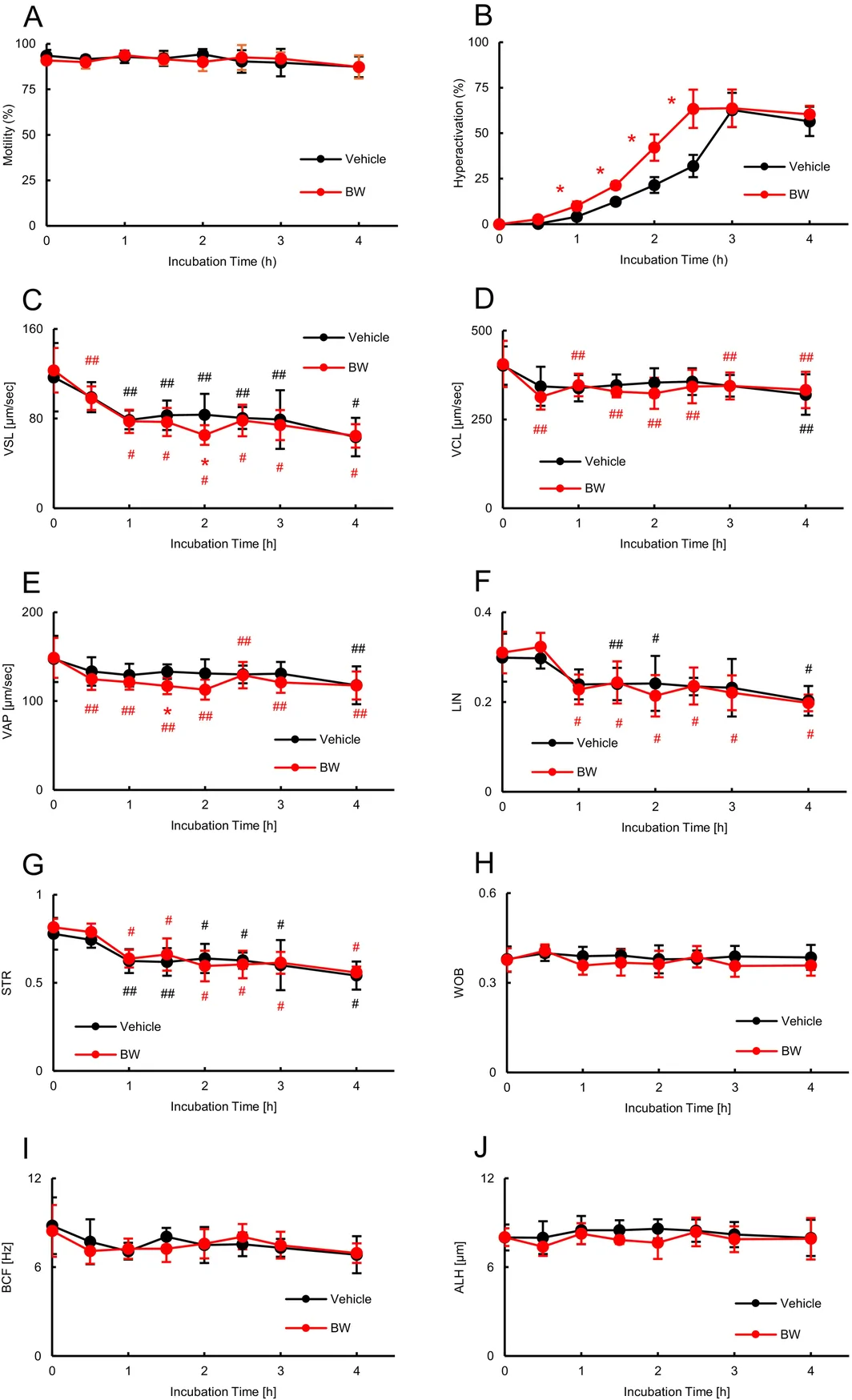
Effects of BW723C86 (BW) on spermatozoal motility, hyperactivation, and motility kinematics. Percentages of motility (A) and hyperactivation (B) are shown when spermatozoa were cultured for 4 h in the presence or absence of BW. Experiments were repeated four times using spermatozoa from four different male mice. As for motility kinematics, straight‐line velocity (VSL) (C), curvilinear velocity (VCL) (D), average‐path velocity (VAP) (E), linearity (LIN) (F), straightness (STR) (G), wobble coefficient (WOB) (H), beat‐cross frequency (BCF) (I), and amplitude of lateral head displacement (ALH) (J) are shown when spermatozoa were cultured for 4 h in the presence or absence of BW. Motility kinematic analyses were repeated six times using spermatozoa from six different male mice. Data represent the mean ± standard deviation. (A–J): Medium containing 0.1% dimethyl sulfoxide as the vehicle; (BW) medium containing 2 μM BW and vehicle. *Significant differences compared with Vehicle in the same time (*p* < 0.01). ^#^Significant differences compared with 0 and 0.5 h (*p* < 0.01). ^##^Significant differences compared with 0 h (*p* < 0.01).

**FIGURE 7:**
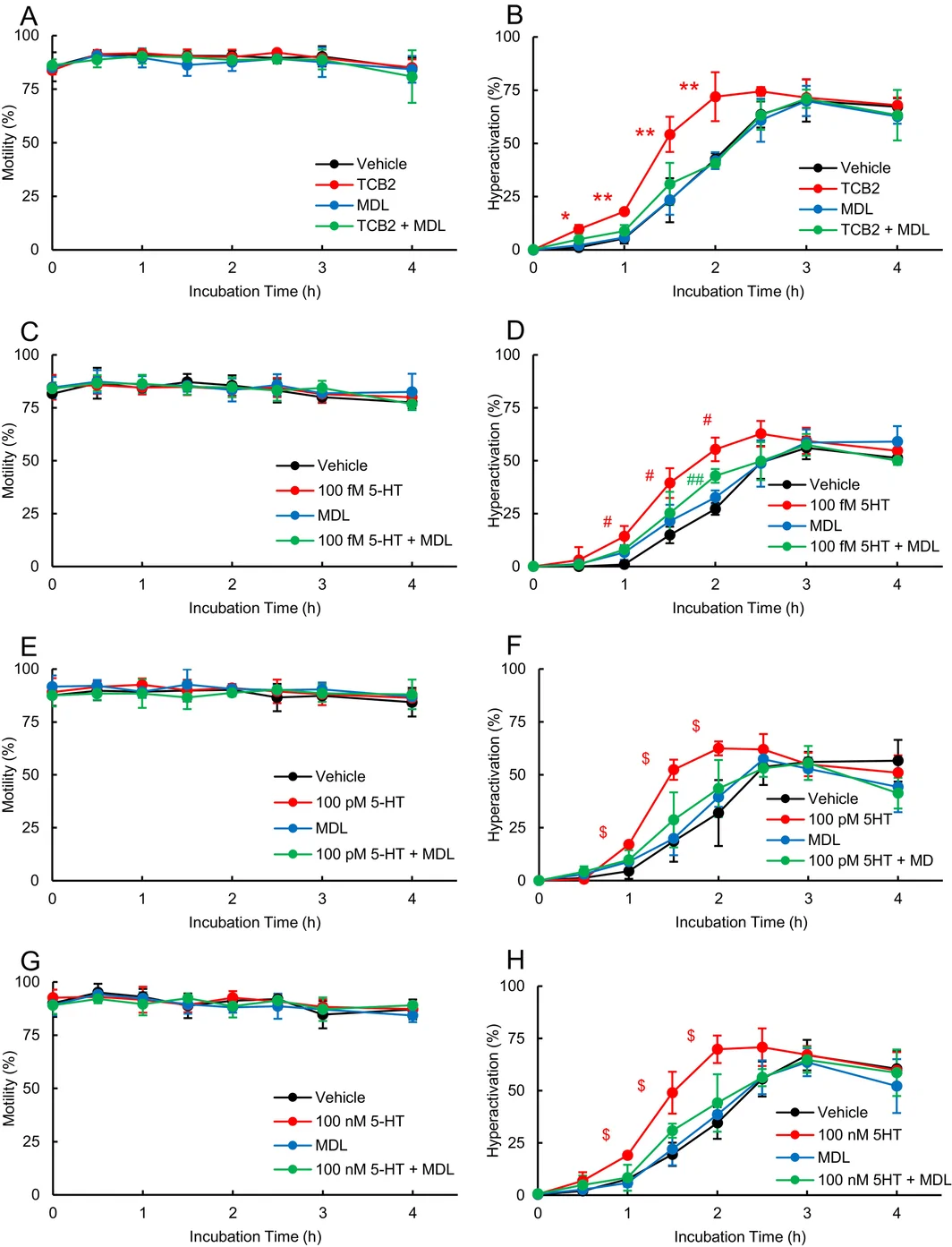
Involvement of the 5‐HT_2A_ receptor in spermatozoal motility and hyperactivation. Percentages of motility (A, C, E, and G) and hyperactivation (B, D, F, and H) are shown when spermatozoa were cultured for 4 h in the presence or absence of 5‐hydroxytryptamine (5‐HT), TCB2, and MDL11939 (MDL). Experiments were repeated four times using spermatozoa from four different male mice. Data represent the mean ± standard deviation. (A and B) medium containing 0.1% pure water and 0.1% dimethyl sulfoxide (DMSO) as the vehicle; (TCB2) medium containing 750 pM TCB2 and vehicle; (MDL) medium containing 540 pM MDL and vehicle; (TCB2 + MDL) medium containing 750 pM TCB2, 540 pM MDL, and vehicle. (C and D) medium containing 0.1% pure water and 0.1% DMSO as the vehicle; (100 fM 5‐HT) medium containing 100 fM 5‐HT and vehicle; (MDL) medium containing 540 pM MDL and vehicle; (100 fM 5‐HT + MDL) medium containing 100 fM 5‐HT, 540 pM MDL, and vehicle. (E and F) medium containing 0.1% pure water and 0.1% DMSO as the vehicle; (100 pM 5‐HT) medium containing 100 pM 5‐HT and vehicle; (MDL) medium containing 540 pM MDL and vehicle; (100 pM 5‐HT + MDL) medium containing 100 pM 5‐HT, 540 pM MDL, and vehicle. (G and H) medium containing 0.1% pure water and 0.1% DMSO as the vehicle; (100 nM 5‐HT) medium containing 100 nM 5‐HT and vehicle; (MDL) medium containing 540 pM MDL and vehicle; (100 nM 5‐HT + MDL) medium containing 100 nM 5‐HT, 540 pM MDL, and vehicle. *Significant differences compared with Vehicle and MDL (*p* < 0.01). **Significant differences compared with Vehicle, MDL, and TCB2 + MDL (*p* < 0.01). ^#^Significant differences compared with Vehicle (*p* < 0.01). ^##^Significant differences compared with Vehicle, 5‐HT, and MDL (*p* < 0.01). ^$^Significant differences compared with Vehicle, MDL, and 5‐HT + MDL (*p* < 0.01).

**FIGURE 8:**
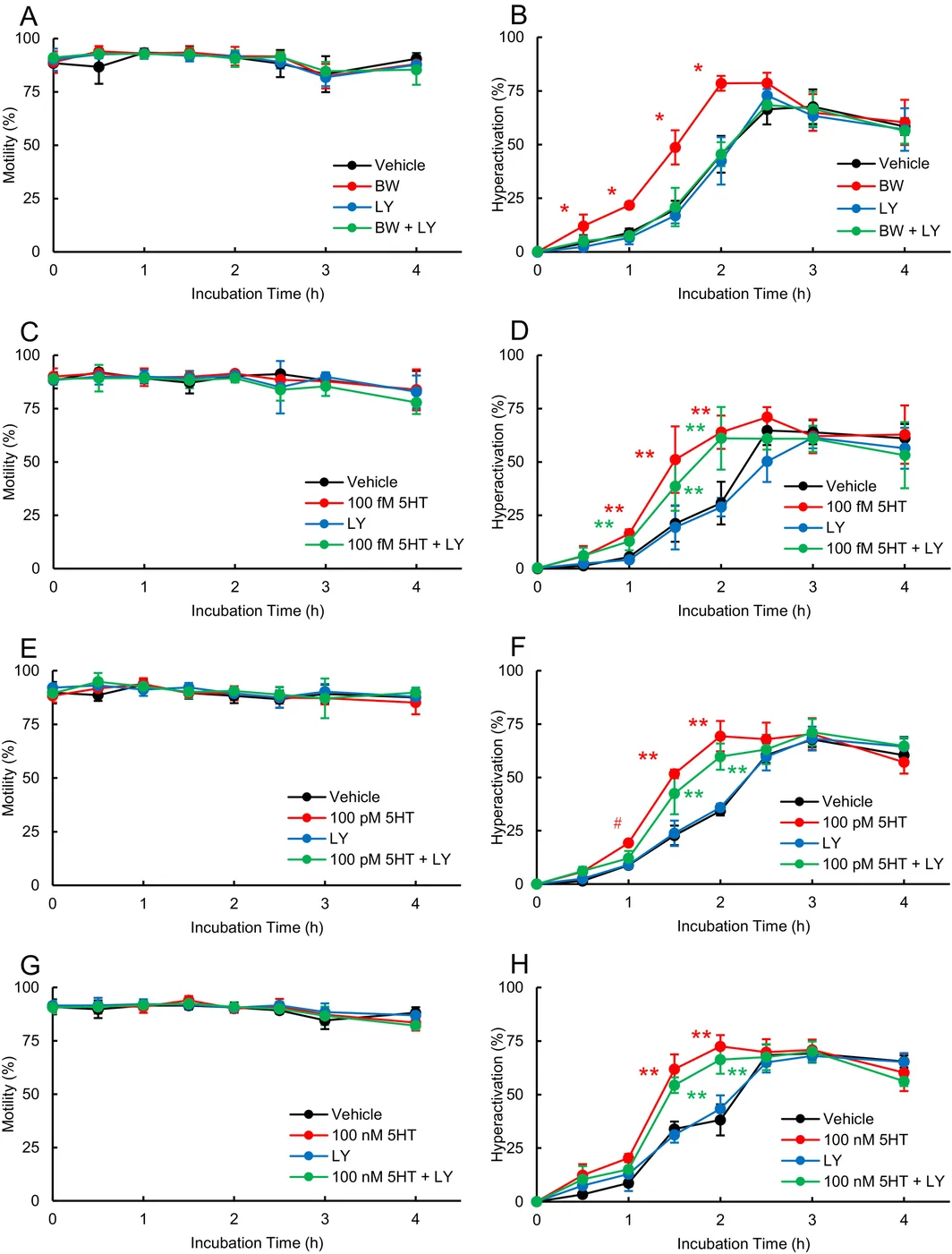
Involvement of the 5‐HT_2B_ receptor in spermatozoal motility and hyperactivation. Percentages of motility (A, C, E, and G) and hyperactivation (B, D, F, and H) are shown when spermatozoa were cultured for 4 h in the presence or absence of 5‐HT, BW723C86 (BW), and LY272015 (LY). Experiments were repeated four times using spermatozoa from four different male mice. Data represent the mean ± standard deviation. (A and B) medium containing 0.2% dimethyl sulfoxide (DMSO) as the vehicle; (BW) medium containing 2 μM BW and vehicle; (LY) medium containing 750 pM LY and vehicle; (BW + LY) medium containing 2 μM BW, 750 pM LY, and vehicle. (C and D) medium containing 0.2% DMSO as the vehicle; (100 fM 5‐HT) medium containing 100 fM 5‐HT and vehicle; (LY) medium containing 750 pM LY and vehicle; (100 fM 5‐HT + LY) medium containing 100 fM 5‐HT, 750 pM LY, and vehicle. (E and F) medium containing 0.2% DMSO as the vehicle; (100 pM 5‐HT) medium containing 100 pM 5‐HT and vehicle; (LY) medium containing 750 pM LY and vehicle; (100 pM 5‐HT + LY) medium containing 100 pM 5‐HT, 750 pM LY, and vehicle. (G and H) medium containing 0.2% DMSO as the vehicle; (100 nM 5‐HT) medium containing 100 nM 5‐HT and vehicle; (LY) medium containing 750 pM LY and vehicle; (100 nM 5‐HT + LY) medium containing 100 nM 5‐HT, 750 pM LY, and vehicle. *Significant differences compared with Vehicle, LY, and BW + LY (*p* < 0.01). **Significant differences compared with Vehicle and LY (*p* < 0.01). ^#^Significant differences compared with Vehicle, LY, and 5‐HT + LY (*p* < 0.01).

Data were presented as mean ± standard deviation. In Figures 1, 2, 3, 4, 5, 6, Tables 1 and 3A,C, and Tables S1 and S2, significant differences between vehicle and agonist were analyzed using Student's *t*‐test in Microsoft Excel (Microsoft Japan, Tokyo, Japan). In Figures 1, 2, 3, 4, 5, 6, significant differences among time‐dependent effects on motility kinematics were analyzed using one‐way analysis of variance and a post hoc test (Student–Newman–Keuls test) in Microsoft Excel with ystat2018 (Igakutosho Shuppan, Saitama, Japan). In Figures 7 and 8, Tables 2 and 3B,D, and Figure S2, significant differences among four groups were analyzed using one‐way analysis of variance and a post hoc test (Student–Newman–Keuls test) in Microsoft Excel with ystat2018. Statistical significance was set at *p* < 0.01.

**TABLE 1:** Effects of 5‐HT_2_ receptor agonists on the IVF success.

	No. of eggs	No. of two‐cell embryos	Two‐cell embryos (%)
5 h insemination			
A			
Vehicle (H_2_O)	87	48	53.00 ± 14.42
MS	66	42	62.56 ± 22.99
B			
Vehicle (H_2_O)	97	56	59.00 ± 18.81
TCB2	75	40	52.51 ± 12.96
C			
Vehicle (DMSO)	73	36	56.08 ± 20.18
BW	107	53	54.27 ± 23.61
0.5 h insemination			
D			
Vehicle (H_2_O)	94	44	46.75 ± 11.06
MS	64	49	74.87 ± 9.81*
E			
Vehicle (H_2_O)	102	46	43.73 ± 6.61
TCB2	72	47	66.45 ± 6.59*
F			
Vehicle (DMSO)	93	41	47.63 ± 9.54
BW	86	60	71.97 ± 11.71*

*Note:* Spermatozoa and COCs were co‐incubated for 0.5 and 5 h. Data indicate the mean ± standard deviation. (A and D) (Vehicle [H_2_O]): 0.1% (v/v) pure water; (MS): 100 fM MS and 0.1% (v/v) pure water. (B and E) (Vehicle [H_2_O]): 0.1% (v/v) pure water; (TCB2): 750 pM TCB2 and 0.1% (v/v) pure water. (C and F) (Vehicle [DMSO]): 0.1% (v/v) DMSO; (BW): 2 μM BW and 0.1% (v/v) DMSO.

Abbreviations: BW, BW723C86; DMSO, dimethyl sulfoxide; IVF, in vitro fertilization; MS, α‐methylserotonin maleate.

* Significant differences compared with “Vehicle (H_2_O)” or “Vehicle (DMSO)” (*p* < 0.01).

**TABLE 2:** Effects of 5‐HT_2_ receptor agonists and antagonists on the IVF success.

	No. of eggs	No. of two‐cell embryos	Two‐cell embryos (%)
0.5 h insemination			
A			
Vehicle (H_2_O and Ethanol)	140	65	47.16 ± 5.87
MS	144	103	71.42 ± 2.65*
Cypro	108	50	44.60 ± 7.91
MS + Cypro	59	31	52.32 ± 11.94
B			
Vehicle (H_2_O and Ethanol)	111	47	43.23 ± 8.80
TCB2	81	63	78.25 ± 7.16*
Cypro	143	65	45.04 ± 5.40
TCB2 + Cypro	84	42	48.96 ± 4.99
C			
Vehicle (H_2_O and DMSO)	129	60	45.08 ± 8.55
TCB2	124	103	82.76 ± 15.42*
MDL	141	70	50.12 ± 5.39
TCB2 + MDL	91	46	53.57 ± 8.88
D			
Vehicle (DMSO and Ethanol)	125	55	42.68 ± 4.28
BW	107	73	66.26 ± 14.60 *
Cypro	102	44	42.45 ± 6.03
BW + Cypro	106	49	45.87 ± 5.15
E			
Vehicle (DMSO)	126	61	47.81 ± 12.40
BW	84	66	80.85 ± 12.07*
LY	94	40	44.16 ± 6.85
BW + LY	79	41	53.13 ± 6.99

*Note:* Spermatozoa and COCs were co‐incubated for 0.5 h. Data indicate the mean ± standard deviation. (A) (Vehicle [H_2_O and Ethanol]): 0.1% (v/v) pure water and 0.1% (v/v) ethanol as vehicle; (MS): 100 fM MS and vehicle; (Cypro): 1 μM Cypro and vehicle; (MS + Cypro): 100 fM MS, 1 μM Cypro, and vehicle. (B) (Vehicle [H_2_O and Ethanol]): same as above; (TCB2): 750 pM TCB2 and vehicle; (Cypro): 1 μM Cypro and vehicle; (TCB2 + Cypro): 750 pM TCB2, 1 μM Cypro, and vehicle. (C) (Vehicle [H_2_O and DMSO]): 0.1% (v/v) pure water and 0.1% (v/v) DMSO as vehicle; (TCB2): 750 pM TCB2 and vehicle; (MDL): 540 pM MDL and vehicle; (TCB2 + MDL): 750 pM TCB2, 540 pM MDL, and vehicle. (D) (Vehicle [DMSO and Ethanol]): 0.1% (v/v) DMSO and 0.1% (v/v) ethanol as vehicle; (BW): 2 μM BW and vehicle; (Cypro): 1 μM Cypro and vehicle; (BW + Cypro): 2 μM BW, 1 μM Cypro, and vehicle. (E) (Vehicle [DMSO]): 0.2% (v/v) DMSO as vehicle; (BW): 2 μM BW and vehicle; (LY): 750 pM LY and vehicle; (BW + LY): 2 μM BW, 750 pM LY and vehicle.

Abbreviations: BW, BW723C86; DMSO, dimethyl sulfoxide; IVF, in vitro fertilization; LY, LY272015; MDL, MDL11939; MS, α‐methylserotonin maleate.

* Significant differences compared with “Vehicle,” “Antagonist,” and “Agonist + Antagonist” (*p* < 0.01).

**TABLE 3:** Effects of 5‐HT_4_ receptor agonists and antagonists on the IVF success.

	No. of eggs	No. of two‐cell embryos	Two‐cell embryos (%)
5 h insemination			
A			
Vehicle (Ethanol)	111	42	40.60 ± 9.14
MT	134	96	72.43 ± 17.48*
B			
Vehicle (Ethanol + DMSO)	79	39	48.83 ± 7.88
MT	56	43	77.40 ± 10.67**
GR	72	35	43.36 ± 11.81
MT + GR	76	41	52.68 ± 8.95
0.5 h insemination			
C			
Vehicle (Ethanol)	111	50	46.59 ± 12.20
MT	115	79	68.21 ± 5.57*
D			
Vehicle (Ethanol + DMSO)	107	46	46.33 ± 3.54
MT	104	76	78.72 ± 9.45**
GR	103	49	46.79 ± 9.16
MT + GR	141	63	45.05 ± 5.61

*Note:* Spermatozoa and COCs were co‐incubated for 0.5 and 5 h. Data indicate the mean ± standard deviation. (A and C) (Vehicle [Ethanol]): 0.1% (v/v) ethanol; (MT): 10 pM MT and vehicle. (B and D) (Vehicle [Ethanol + DMSO]): 0.1% (v/v) ethanol and 0.1% (v/v) DMSO; (MT): 10 pM MT and vehicle; (GR): 1 μM GR and vehicle; (MT + GR): 10 pM MT, 1 μM GR, and vehicle.

Abbreviations: DMSO, dimethyl sulfoxide; GR, GR113808; IVF, in vitro fertilization; MT, 5‐methoxytryptamine.

* Significant differences compared with “Vehicle (Ethanol)” (*p* < 0.01).

** Significant differences compared with “Vehicle (Ethanol),” “GR,” and “MT + GR” (*p* < 0.01).

## 3 Results

### 3.1 Time Dependent Effects of 5‐HT Agonists on Motility, Hyperactivation, and Motility Kinematics

Our previous study [6] showed that 5‐HT enhanced hyperactivation through 5‐HT_2_, 5‐HT_3_, 5‐HT_4_, and 5‐HT_7_ receptors. As shown in Figure S1 lanes c, d, k, and l, we detected 5‐HT_2A_ and 5‐HT_3B_ receptors from the urea extract and the urea‐thiourea extract of mouse spermatozoa. 5‐HT_2B_, 5‐HT_2C_, 5‐HT_3A_, 5‐HT_4_, and 5‐HT_7_ receptors were detected from the urea extract (Figure S1 lanes e–j,m–p).

In the next step, we examined the effects of each 5‐HT receptor agonist on spermatozoal motility, hyperactivation, and motility kinematics. As shown in Figure 1A,B, 100 fM MS, which is the 5‐HT_2_ receptor agonist [instruction manual] [4], significantly enhanced hyperactivation at incubation for 1, 1.5, and 2 h, although it did not affect motility. Moreover, the motility kinematics of spermatozoa exposed to 100 fM MS were analyzed (Figure 1C–J). VSL was reduced at incubation for 0.5, 1, 1.5, 2, 2.5, 3, and 4 h compared with incubation for 0 h in the presence of MS, whereas it was reduced at incubation for 1, 1.5, 2, 2.5, and 3 h compared with incubation for 0 h in the absence of MS (Figure 1C). VCL was reduced at incubation for 1, 1.5, 2, 2.5, 3, and 4 h compared with incubation for 0 h in the presence of MS, whereas it was reduced at incubation for 2.5 and 3 h compared with incubation for 0 h in the absence of MS (Figure 1D). VAP was reduced at incubation for 3 h compared with incubation for 0 h in the presence of MS and at incubation for 3 and 4 h compared with incubation for 0 h in the absence of MS (Figure 1E). LIN was reduced at incubation for 1.5, 2, 2.5, 3, and 4 h compared with incubation for 0 h in the presence of MS, whereas it was reduced at incubation for 1, 1.5, 2, 2.5, 3, and 4 h compared with incubation for 0 h in the absence of MS (Figure 1F). Regardless of the presence of MS, STR was reduced at incubation for 1, 1.5, 2, 2.5, 3, and 4 h compared with incubation for 0 h (Figure 1G). As shown in Figure 1H, WOB was increased at incubation for 2 and 2.5 h compared with incubation for 0 h in the presence of MS. BCF was reduced at incubation for 0.5 and 1.5 h compared with incubation for 0 h in the presence of MS, although it was reduced at incubation for 0.5, 1, 1.5, 2, 2.5, 3, and 4 h compared with incubation for 0 h in the absence of MS (Figure 1I). ALH increased at incubation for 1 and 1.5 h compared with incubation for 0 h in the presence of MS, although it increased at incubation for 0.5, 1, 1.5, 2, 2.5, 3, and 4 h compared with incubation for 0 h in the absence of MS (Figure 1J). In addition, MS decreased VSL, VCL, LIN, STR, and ALH at incubation for 2 h compared with the vehicle (Figure 1C,D,F,G,J). MS also decreased BCF at incubation for 0.5 h compared with the vehicle (Figure 1I).

As shown in Figure 2A,B, 100 μM mCPBG, which is a 5‐HT_3_ receptor agonist [29], significantly enhanced hyperactivation at incubation for 2 h although it did not affect motility. In addition, motility kinematics of spermatozoa exposed to 100 μM mCPBG were analyzed (Figure 2C–J). VSL was reduced at incubation for 1, 1.5, 2, 2.5, 3, and 4 h compared with incubation for 0 h regardless of the presence of mCPBG (Figure 2C). LIN was reduced at incubation for 3 h compared with incubation for 0 h in the presence of mCPBG, and it was reduced at incubation for 2.5, 3, and 4 h compared with incubation for 0 h in the absence of mCPBG (Figure 2F). STR was reduced at incubation for 3 h compared with incubation for 0 h in the presence of mCPBG and it was reduced at incubation for 2, 2.5, 3, and 4 h compared with incubation for 0 h in the absence of mCPBG (Figure 2G). BCF was reduced at incubation for 1, 1.5, 2, 2.5, 3, and 4 h compared with incubation for 0 h in the presence of mCPBG and it was reduced at incubation for 0.5, 1, 1.5, 2, 2.5, 3, and 4 h compared with incubation for 0 h in the absence of mCPBG (Figure 2I). At incubation for 2 h, mCPBG decreased VSL, LIN, WOB, and BCF compared with the vehicle (Figure 2C,F,H,I). In contrast, mCPBG did not affect VCL, VAP, and ALH (Figure 2D,E,J).

MT at 10 pM, which is the 5‐HT_4_ receptor agonist [instruction manual] [4, 30], significantly enhanced hyperactivation at incubation for 1, 1.5, and 2 h, although it did not affect motility (Figure 3A,B). Effects of 10 pM MT on motility kinematics were examined (Figure 3C–J). VSL, VCL, and BCF were reduced at incubation for 0.5, 1, 1.5, 2, 2.5, 3, and 4 h compared with incubation for 0 h in the presence of MT, and they were reduced at incubation for 1, 1.5, 2, 2.5, 3, and 4 h compared with incubation for 0 h in the absence of MT (Figure 3C,D,I). VAP was reduced at incubation for 0.5, 1, 1.5, 2, 2.5, 3, and 4 h compared with incubation for 0 h in the presence of MT, and it was reduced at incubation for 2, 2.5, 3, and 4 h compared with incubation for 0 h in the absence of MT (Figure 3E). Regardless of the presence of MT, LIN and STR were reduced at incubation for 1, 1.5, 2, 2.5, 3, and 4 h compared with incubation for 0 h (Figure 3F,G). As shown in Figure 3H, WOB was increased at incubation for 2 h compared with incubation for 0 h in the presence of MT. Moreover, MT reduced VCL and ALH but increased WOB at incubation for 2 h compared with the vehicle (Figure 3D,H,J).

LP12 at 130 pM, which the 5‐HT_7_ receptor agonist [instruction manual], significantly enhanced hyperactivation at incubation for 2 h, although it did not affect motility (Figure 4A,B). As shown in Figure 4C–J, motility kinematics of spermatozoa exposed to 130 pM LP12 were analyzed. In the absence of LP12, VSL, and LIN were reduced at incubation for 2.5, 3, and 4 h compared with incubation for 0 h (Figure 4C,F). VAP was reduced at incubation for 2 h compared with incubation for 0 h in the presence of LP12 and at incubation for 4 h compared with incubation for 0 h in the absence of LP12 (Figure 4E). As shown in Figure 4G, STR was reduced at incubation for 2.5, 3, and 4 h compared with incubation for 0 h regardless of the presence of LP12. BCF was reduced at incubation for 4 h compared with incubation for 0 h regardless of the presence of LP12 (Figure 4I). LP12 did not affect VCL, WOB, or ALH (Figure 4D,H,J).

Because it was reported that MT non‐selectively stimulates 5‐HT receptors [31], we examined whether MT‐enhanced hyperactivation was suppressed by antagonists of 5‐HT_2_, 5‐HT_3_, 5‐HT_4_, and 5‐HT_7_ receptors (Figure S2). Cypro (5‐HT_2_ receptor antagonist) [instruction manual], DS (5‐HT_3_ receptor antagonist) [32], and SB (5‐HT_7_ receptor antagonist) [33] did not suppress MT‐enhanced hyperactivation (Figure 2B,D,H). However, GR (5‐HT_4_ receptor antagonist) [instruction manual] [34] suppressed MT‐enhanced hyperactivation (Figure 2F). Theses 5‐HT receptor antagonists did not affect motility (Figure 2A,C,E,G).

### 3.2 Involvement of 5‐HT_2_ Receptor Subtypes in Motility, Hyperactivation, and Motility Kinematics

Because the 5‐HT_2_ receptor consists of three subtypes, we examined which subtypes were associated with the enhancement of mouse spermatozoal hyperactivation. Three 5‐HT_2_ receptor subtypes were detected from mouse spermatozoa (Figure S1). As shown in Figure 5A,B, 750 pM TCB2, which is the 5‐HT_2A_ receptor agonist [instruction manual] [35], significantly enhanced hyperactivation at incubation for 1, 1.5, 2, and 2.5 h, although it did not affect motility. As shown in Figure 5C–J, motility kinematics of spermatozoa exposed to 750 pM TCB2 were analyzed. In the presence of TCB2, VSL was significantly reduced at incubation for 0.5, 1, 1.5, 2, 2.5, 3, and 4 h compared with incubation for 0 h, and it was reduced at incubation for 1.5, 2, 2.5, 3, and 4 h compared with incubation for 0 h in the absence of TCB2 (Figure 5C). Regardless of the presence of TCB2, VCL was reduced at incubation for 0.5, 1, 1.5, 2, 2.5, 3, and 4 h compared with incubation for 0 h (Figure 5D). VAP was reduced at incubation for 0.5, 1, 1.5, 2, 2.5, 3, and 4 h compared with incubation for 0 h in the presence of TCB2, and it was reduced at incubation for 2.5, 3, and 4 h compared with incubation for 0 h in the absence of TCB2 (Figure 5E). LIN was reduced at incubation for 1.5, 2, 2.5, 3, and 4 h compared with incubation for 0 h regardless of the presence of TCB2 (Figure 5F). STR was reduced at incubation for 1.5, 2, 2.5, 3, and 4 h compared with incubation for 0 h in the presence of TCB2, and it was reduced at incubation for 2, 2.5, 3, and 4 h compared with incubation for 0 h in the absence of TCB2 (Figure 5G). BCF was reduced at incubation for 0.5, 1, 1.5, 2, 2.5, 3, and 4 h compared with incubation for 0 h in the absence of TCB2 (Figure 5I). In addition, TCB2 significantly reduced VSL, VCL, VAP, and ALH at incubation for 1, 1.5, and 2 h compared with the vehicle (Figure 5C–E,J). TCB2 did not affect WOB (Figure 5H).

BW at 2 μM, which is the 5‐HT_2B_ receptor agonist [35], significantly enhanced hyperactivation at incubation for 1, 1.5, 2, and 2.5 h, whereas it did not affect motility (Figure 6A,B). As shown in Figure 6C–J, motility kinematics of spermatozoa exposed to 2 μM BW were analyzed. VSL was reduced at incubation for 0.5, 1, 1.5, 2, 2.5, 3, and 4 h compared with incubation for 0 h in the presence of BW, and it was reduced at incubation for 1, 1.5, 2, 2.5, 3, and 4 h compared with incubation for 0 h in the absence of BW (Figure 6C). VCL and VAP were reduced at incubation for 0.5, 1, 1.5, 2, 2.5, 3, and 4 h compared with incubation for 0 h in the presence of BW, and they were reduced at incubation for 4 h compared with incubation for 0 h in the absence of BW (Figure 6D,E). LIN was reduced at incubation for 1, 1.5, 2, 2.5, 3, and 4 h compared with incubation for 0 h in the presence of BW, and it was reduced at incubation for 1.5, 2, and 4 h compared with incubation for 0 h in the absence of BW (Figure 6F). STR was reduced at incubation for 1, 1.5, 2, 2.5, 3, and 4 h compared with incubation for 0 h regardless of the presence of BW (Figure 6G). BW did not affect WOB, BCF, or ALH (Figure 6H–J).

MK212 at 300 pM, which is the 5‐HT_2C_ receptor agonist [instruction manual], did not affect motility and hyperactivation (Figure S3).

### 3.3 Dose‐Dependent Effects of 5‐HT on 5‐HT_2A_ and 5‐HT_2B_ Receptors

We examined effects of 5‐HT on 5‐HT_2A_ and 5‐HT_2B_ receptors because 5‐HT_2A_ and 5‐HT_2B_ receptor agonists enhanced hyperactivation (Figures 5B and 6B). As shown in Figure 7A,B, TCB2‐enhanced hyperactivation was suppressed by 540 pM MDL, which is a 5‐HT_2A_ receptor antagonist [instruction manual], although motility was not affected. 5‐HT at 100 fM, 100 pM, and 100 nM enhanced hyperactivation at incubation for 1, 1.5, and 2 h (Figure 7D,F,H). Although hyperactivation enhanced by 100 fM 5‐HT was not suppressed by MDL at incubation for 1 and 1.5 h, the enhancement was suppressed by MDL at incubation for 2 h (Figure 7D). Moreover, suppression of 100 fM 5‐HT‐enhanced hyperactivation by MDL at incubation for 2 h was midway between the vehicle and 100 fM 5‐HT. As shown in Figure 7F,H, hyperactivation enhanced by 100 pM and 100 nM 5‐HT was suppressed by MDL. 5‐HT and MDL did not affect motility (Figure 7C,E,G).

As shown in Figure 8A,B, BW‐enhanced hyperactivation was suppressed by 750 pM LY, which is a 5‐HT_2B_ receptor antagonist [instruction manual] [35], although motility was not affected. 5‐HT at 100 fM and 100 pM enhanced hyperactivation at incubation for 1, 1.5, and 2 h (Figure 8D,F). Hyperactivation enhanced by 100 fM 5‐HT was not suppressed by LY (Figure 8D). As shown in Figure 8F, hyperactivation enhanced by 100 pM 5‐HT was slightly suppressed by LY at incubation for 1 h, although the enhancement was not suppressed by LY at incubation for 1.5 and 2 h. As shown in Figure 8H, LY did not suppress 100 nM 5‐HT‐enhanced hyperactivation. 5‐HT and LY did not affect motility (Figure 8C,E,G).

### 3.4 Effects of 5‐HT Receptor Agonists on IVF Success

As shown in Table 1, MS, TCB2, and BW increased IVF success when spermatozoa and COCs were co‐incubated for 0.5 h, although they did not affect IVF success when spermatozoa and COCs were co‐incubated for 5 h. The increases in IVF success induced by MS, TCB2, and BW were suppressed by Cypro, MDL, and LY (Table 2). As shown in Table 3, MT increased IVF success when spermatozoa and COCs were co‐incubated for 0.5 or 5 h. Moreover, the effects of MT on IVF success were suppressed by GR, which is a 5‐HT_4_ receptor antagonist. In contrast, mCPBG and LP12 did not affect IVF success when spermatozoa and COCs were co‐incubated for 0.5 or 5 h (Tables S1 and S2).

## 4 Discussion

In rodent spermatozoa, 5‐HT is one of the effectors that induce capacitation [3, 4, 6, 7]. In hamsters, a high concentration of 5‐HT induced the AR and enhanced hyperactivation through the 5‐HT_4_ receptor [3, 4]. When hamster spermatozoa were exposed to a low concentration of 5‐HT, their hyperactivation was enhanced through the 5‐HT_2_ receptor [4]. Moreover, hamster spermatozoa synthesize 5‐HT from tryptophan and are hyperactivated through stimulation of the 5‐HT_4_ receptor [14]. In rats, 5‐HT enhanced hyperactivation and increased IVF success through the 5‐HT_4_ receptor [7]. In mice, 5‐HT enhanced hyperactivation through 5‐HT_2_, 5‐HT_3_, 5‐HT_4_, and 5‐HT_7_ receptors and increased IVF success through the 5‐HT_4_ receptor [6]. In the present study, we detected 5‐HT_2_, 5‐HT_3_, 5‐HT_4_, and 5‐HT_7_ receptors from mouse spermatozoa (Figure S1). Moreover, we examined the effects of 5‐HT receptor agonists on hyperactivation and IVF success in mice. Agonists of the 5‐HT_2_ and 5‐HT_4_ receptors strongly enhanced hyperactivation, whereas agonists of the 5‐HT_3_ and 5‐HT_7_ receptors weakly enhanced hyperactivation (Figures 1B, 2B, 3B and 4B). Moreover, agonists of the 5‐HT_2A_ and 5‐HT_2B_ receptors enhanced hyperactivation, whereas the 5‐HT_2C_ receptor agonist did not affect it (Figures 5B and 6B, and Figure S3). The 5‐HT_2A_ receptor antagonist suppressed 100 pM and 100 nM 5‐HT‐enhanced hyperactivation, although it slightly suppressed 100 fM 5‐HT‐enhanced hyperactivation (Figure 7D,F,H). The 5‐HT_2B_ receptor antagonist did not usually affect 5‐HT‐enhanced hyperactivation, although it slightly suppressed 100 pM 5‐HT‐enhanced hyperactivation after incubation for 1 h only (Figure 8D,F,H). These present results show that 5‐HT usually enhances mouse spermatozoal hyperactivation through the 5‐HT_2A_ and 5‐HT_4_ receptors. Moreover, these results show that a high concentration of 5‐HT stimulates the 5‐HT_2A_ receptor.

Mouse spermatozoal motility kinematics were weakly affected by 5‐HT and 5‐HT receptor agonists when spermatozoa were incubated for 2 h [6]. In the present study, we analyzed time dependent effects of 5‐HT receptor agonists on mouse motility kinematics when mouse spermatozoa were incubated for 4 h to induce hyperactivation (Figures 1, 2, 3, 4, 5, 6). The 5‐HT_2_, 5‐HT_2A_, 5‐HT_2B_, and 5‐HT_4_ receptor agonists accelerated or enhanced the decrease of VSL and VCL rather than vehicle when they enhanced hyperactivation (Figures 1B–D, 3B–D, 5B–D and 6B–D). In contrast, the 5‐HT_3_ and 5‐HT_7_ receptor agonists weakly enhanced hyperactivation and did not affect the decrease of VSL and VCL (Figures 2B–D and 4B–D). As for VAP, the 5‐HT_2A_, 5‐HT_2B_, and 5‐HT_4_ receptor agonists accelerated or enhanced it rather than vehicle when they enhanced hyperactivation (Figures 3B,E, 5B,E and 6B,E). However, the 5‐HT_2_, 5‐HT_3_, and 5‐HT_7_ receptor no or slightly reduced VAP (Figures 2E, 3E and 4E). LIN, STR, and WOB are defined as VSL/VCL, VSL/VAP, and VAP/VCL [24]. The 5‐HT_2_ receptor agonist delayed the reduction of LIN although it accelerated the reduction of VSL and VCL (Figure 1C,D,F). In addition, the 5‐HT_2_ receptor agonist showed no or slightly affected STR and WOB although it accelerated the reduction of VCL (Figure 1D,G,H). The 5‐HT_2A_ receptor agonist accelerated STR and did not affect LIN and WOB, although it accelerated the reduction of VSL, VCL, and VAP (Figure 5C–H). The 5‐HT_2B_ receptor agonist accelerated the reduction of LIN because it accelerated the reduction of VSL and VCL (Figure 6C,D,F). In contrast, the 5‐HT_2B_ receptor agonist did not affect STR and WOB although it accelerated the reduction of VSL, VCL, and VAP (Figure 6C–E,G,H). The 5‐HT_4_ receptor agonist did not accelerate LIN, STR, and WOB although it accelerated the reduction of VSL, VCL, and VAP (Figure 3C–H). The 5‐HT_3_ and 5‐HT_7_ receptor agonists did not accelerate LIN, STR, and WOB because they did not affect VSL, VCL, and VAP (Figures 2C–H and 5C–H). The 5‐HT_2_, 5‐HT_2A_, 5HT_2B_, and 5‐HT_7_ receptor agonists did not accelerate BCF and ALH (Figures 1I,J, 4I,J, 5I,J and 6I,J). The 5‐HT_3_ receptor agonist delayed the decrease of BCF but did not affect ALH (Figure 2I,J). The 5‐HT_4_ receptor agonist accelerated the decrease of BCF but did not affect ALH (Figure 3I,J). It is likely that some motility kinematics are related to hyperactivation from the effects of 5‐HT on exposure to mouse spermatozoa (Figures 1, 2, 3, 4, 5, 6). However, classifications and characteristics of motility kinematics associated with hyperactivation depend on animal species [24]. In addition, classifications and characteristics of motility kinematics of the same animal are different by animal strains and researches. In general, it is suggested that the common factor of motility kinematics of hyperactivated spermatozoa is the high VCL and ALH and the low LIN or WOB [24]. In the present study, the reduction of VSL was strongly associated with being hyperactivated in mouse spermatozoa (Figures 1, 2, 3, 4, 5, 6). However, other parameters of motility kinematics were not always associated with being hyperactivated. Although we usually analyze spermatozoal movement as two‐dimensional movement, we can not correctly analyze spermatozoal movement because true spermatozoal movement is a complex three‐dimensional movement [36]. Especially, it is difficult to stably measure VCL, VAP, ALH, and BCF because spermatozoal movement shows rotational and whiplash‐like trajectories. Because VSL is the velocity of the linear distance between the start point and end point, measurement of VSL is easier than that of other parameters.

Spermatozoa and oocytes are cultured for 5 h when mouse IVF is conducted according to a commonly used method [28]. Although mouse IVF success was not affected when spermatozoa and oocytes were cultured for 5 h in the presence of 5‐HT, IVF success was increased when spermatozoa and oocytes were cultured for 0.5 h in the presence of 5‐HT [6]. Moreover, the increase in mouse IVF success induced by 5‐HT was suppressed by a 5‐HT_4_ receptor antagonist. In the present study, we examined the effects of 5‐HT receptor agonists on mouse IVF success (Tables 1, 2, 3, and Tables S1 and S2). The 5‐HT_2_, 5‐HT_2A_, and 5‐HT_2B_ receptor agonists increased mouse IVF success when spermatozoa and oocytes were cultured for 0.5 h, but they did not affect mouse IVF success when spermatozoa and oocytes were cultured for 5 h (Tables 1 and 2). The 5‐HT_4_ receptor agonist increased mouse IVF success when spermatozoa and oocytes were cultured for 0.5 and 5 h (Table 3). In contrast, the 5‐HT_3_ and the 5‐HT_7_ receptor agonists did not affect mouse IVF success (Tables S1 and S2). These results suggest that the 5‐HT_2_ and 5‐HT_4_ receptors participate in mouse IVF success. Moreover, the 5‐HT_4_ receptor appears to be more effective than the 5‐HT_2_ receptor. Because the previous study [6] showed that only the 5‐HT_4_ receptor antagonist suppressed the increase in mouse IVF success induced by 5‐HT, it is reasonable that a 5‐HT_4_ receptor agonist increased mouse IVF success and was more effective than other 5‐HT receptors.

Previous [6] and present studies suggest that 5‐HT enhances hyperactivation and increases IVF success in mice. The effects of 5‐HT on hyperactivation and IVF success occur through the 5‐HT_2_ and 5‐HT_4_ receptors. Moreover, the effects of the 5‐HT_4_ receptor are stronger than those of the 5‐HT_2_ receptor. In rats, 5‐HT enhanced hyperactivation and increased IVF success through the 5‐HT_4_ receptor [7]. Hamster spermatozoal hyperactivation was enhanced by 5‐HT through the 5‐HT_2_ and 5‐HT_4_ receptors in a dose‐dependent manner [4]. Moreover, stimulation of the 5‐HT_4_ receptor induced the AR in hamster spermatozoa [3]. After stimulation of the 5‐HT_4_ receptor, tmAC is activated, leading to an increase in cAMP concentration and activation of PKA [2]. In hamster spermatozoa, stimulation of the 5‐HT_4_ receptor activates tmAC and PKA [27]. Moreover, PKA activates the CatSper channel, which subsequently stimulates soluble adenylate cyclase [27]. The CatSper channel is essential for hyperactivation [37]. In mice, the CatSper channel is activated by PKA [38]. A recent study [14] showed that hamster spermatozoa synthesize 5‐HT from tryptophan, and their hyperactivation is enhanced by spermatozoal 5‐HT through the 5‐HT_4_ receptor. Because the 5‐HT_4_ receptor participates in the regulation of AR, hyperactivation, and IVF success in rodents, it appears that the 5‐HT_4_ receptor and its signaling pathways are common for rodent spermatozoal capacitation.

## Funding

This work was supported by JSPS KAKENHI Grant Number JP25K12557 (to M.F.).

## Conflicts of Interest

The authors declare no conflicts of interest.

## Supporting information


**Figure S1:** Detection of 5‐HT receptors from mouse spermatozoa. Lanes (a) and (b) show the Coomassie brilliant blue‐stained gel. Lanes (c) and (d) show the western blotting results using the anti‐5‐HT_2A_ receptor antibody. Lanes (e) and (f) show the western blotting results using the anti‐5‐HT_2B_ receptor antibody. Lanes (g) and (h) show the western blotting results using the anti‐5‐HT_2C_ receptor antibody. Lanes (i) and (j) show the western blotting results using the anti‐5‐HT_3A_ receptor antibody. Lanes (k) and (l) show the western blotting results using the anti‐5‐HT_3B_ receptor antibody. Lanes (m) and (n) show the western blotting results using the anti‐5‐HT_4_ receptor antibody. Lanes (o) and (p) show the western blotting results using the anti‐5‐HT_7_ receptor antibody. Lanes (a, c, e, g, i, k, m, o) show the urea‐extract. Lanes (b, d, f, h, j, l, n, p) show the urea–thiourea extract. Numbers on the left side of lane a represent the molecular weight markers. The volume of the extracts loaded in each lane was 20 μL. Arrowhead indicates the antibody reaction.
**Figure S2:** Effects of 5‐HT_2_, 5‐HT_3_, 5‐HT_4_, and 5‐HT_7_ receptor antagonists on methoxytryptamine (MT) enhanced hyperactivation. Percentages of motility (A, C, E, and G) and hyperactivation (B, D, F, and H) are showed when mouse spermatozoa were exposed to MT after exposure of cyproheptadine (Cypro, 5‐HT_2_ receptor antagonist), dolasetron (DS, 5‐HT_3_ receptor antagonist), GR113808 (GR, 5‐HT_4_ receptor antagonist), and SB‐258719 (SB, 5‐HT_7_ receptor antagonist). Experiments were repeated four times using spermatozoa from four different male mice. Data represent the mean ± standard deviation. (A and B) (Vehicle) medium containing 0.2% ethanol as vehicle; (MT) medium containing 10 pM MT and vehicle; (Cypro) medium containing 1 μM Cypro and vehicle; (MT + Cypro) medium containing 1 pM MT, 1 μM Cypro and vehicle. (C and D) (Vehicle) medium containing 0.1% pure water and 0.1% ethanol as vehicle; (MT) medium containing 10 pM MT and vehicle; (DS) medium containing 3.8 nM DS and vehicle; (MT + DS) medium containing 1 pM MT, 3.8 nM DS and vehicle. (E and F) (Vehicle) medium containing 0.1% ethanol and 0.1% dimethyl sulfoxide as vehicle; (MT) medium containing 10 pM MT and vehicle; (GR) medium containing 1 μM GR and vehicle; (MT + GR) medium containing 1 pM MT, 1 μM GR and vehicle. (G and H) (Vehicle) medium containing 0.1% ethanol and 0.1% dimethyl sulfoxide as vehicle; (MT) medium containing 10 pM MT and vehicle; (SB) medium containing 10 μM SB and vehicle; (MT + SB) medium containing 1 pM MT, 10 μM SB and vehicle. *Significant differences compared with Vehicle and antagonist (*p* < 0.01). **Significant differences compared with Vehicle, antagonist, and MT + antagonist (*p* < 0.01).
**Figure S3:** Effects of MK212 on spermatozoal motility and hyperactivation. Percentages of motility (A) and hyperactivation (B) are shown when spermatozoa were cultured for 4 h in the presence or absence of MK212. Experiments were repeated four times using spermatozoa from four different male mice. Data represent the mean ± standard deviation. (A and B): (Vehicle) medium containing 0.1% pure water as the vehicle; (MK212) medium containing 300 pM MK212 and vehicle.

**Table S1:** Effects of 5‐HT_3_ receptor agonist on the IVF success.
**Table S2:** Effects of 5‐HT_7_ receptor agonists and antagonists on the IVF success.

## Data Availability

Data that support the results reported here are available from the corresponding author, M.F., upon reasonable request.
