# Supplementary material for: Influences of 5‐Hydroxytryptamine Receptor Agonists on Spermatozoal Hyperactivation and In Vitro Fertilization Success in Mice
Source: Reprod Med Biol. 2026 Sep 16;25(1):e70092. doi: 10.1002/rmb2.70092 (PMC13583447; doi:10.1002/rmb2.70092)
Supplement: Supplementary file 1 [file RMB2-25-e70092-s001.pdf]

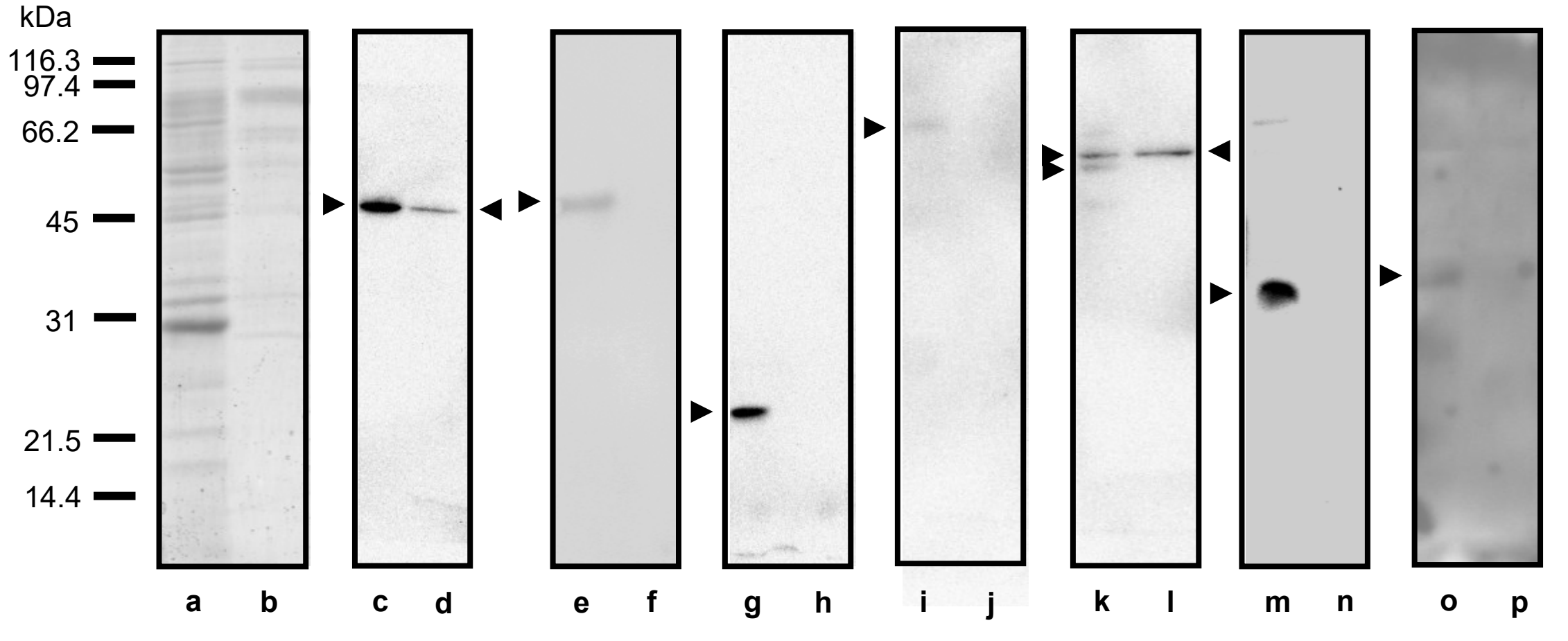

Supplementary Figure 1.

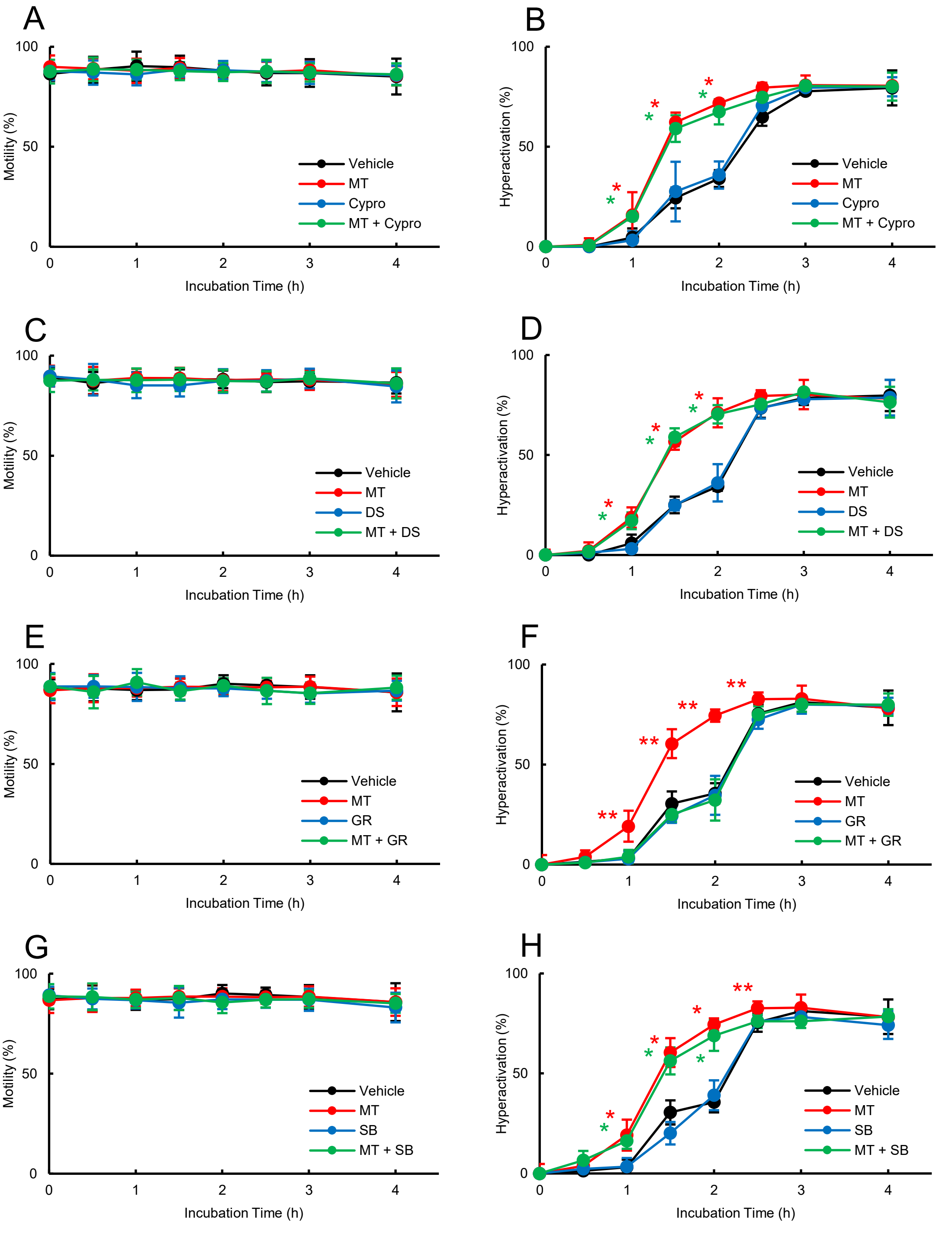

Supplementary Figure 2.

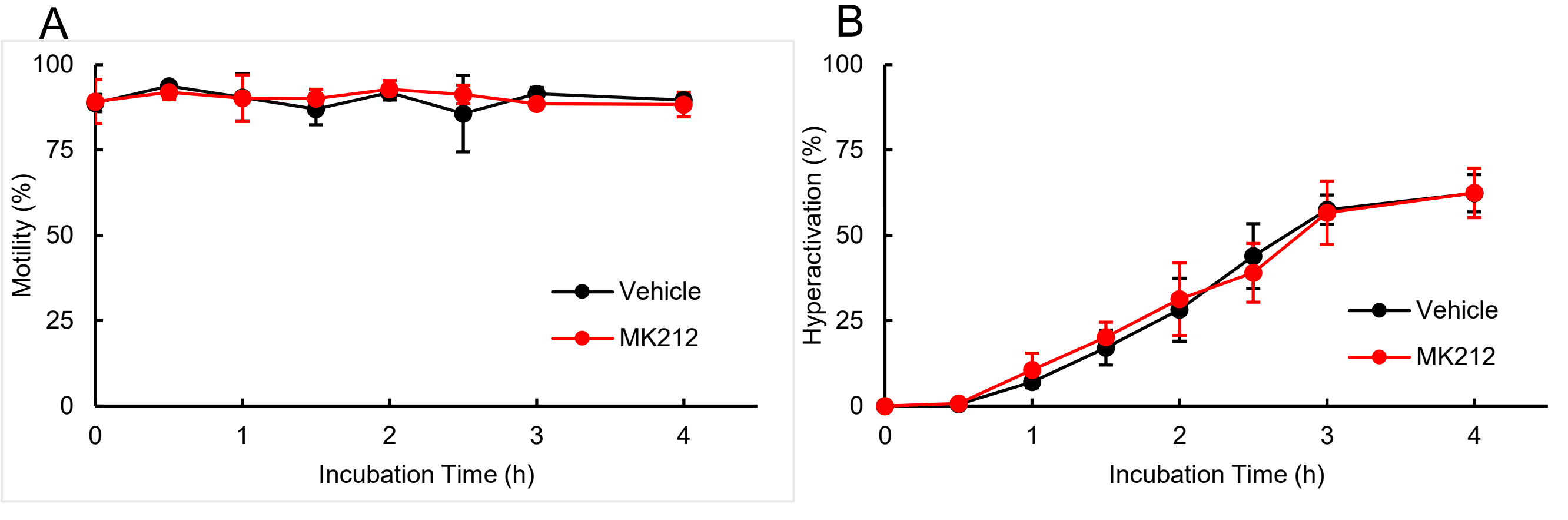

Supplementary Fig. 3
