# Supplementary material for: Influences of 5‐Hydroxytryptamine Receptor Agonists on Spermatozoal Hyperactivation and In Vitro Fertilization Success in Mice
Source: Reprod Med Biol. 2026 Sep 16;25(1):e70092. doi: 10.1002/rmb2.70092 (PMC13583447; doi:10.1002/rmb2.70092)
Supplement: Supplementary file 2 [file RMB2-25-e70092-s002.pdf]

Supplementary Table 1 Effects of 5-HT_3 receptor agonist on the IVF success

	No. of eggs	No. of two-cell embryos	Two-cell embryos (%)
5 h insemination			
Vehicle (H_2O)	57	28	49.23 ± 6.43
mCPBG	96	46	49.34 ± 13.67
0.5 h insemination			
Vehicle (H_2O)	96	46	46.56 ± 7.59
mCPBG	67	31	47.09 ± 13.93

Spermatozoa and COCs were co-incubated for 0.5 and 5 h. Data indicate the mean ± standard deviation. (Vehicle [H_2O]): 0.1% (v/v) pure water; (mCPBG): 100 μ M mCPBG and vehicle. IVF, *in vitro* fertilization; mCPBG, 1-(3-chlorophenyl)biguanide hydrochloride.

Supplementary Table 2 Effects of 5-HT_7 receptor agonists and antagonists on the IVF success

	No. of eggs	f two-cell eml	Two-cell embryos (%)
5 h insemination			
Vehicle (H_2O)	104	58	52.15 ± 21.58
LP12	93	54	52.22 ± 16.43
0.5 h insemination			
Vehicle (H_2O)	128	67	52.05 ± 8.74
LP12	112	75	65.49 ± 6.44

Spermatozoa and COCs were co-incubated for 0.5 and 5 h. Data indicate the mean ± standard deviation. (Vehicle [H_2O]): 0.1% (v/v) pure water; (LP): 130 pM LP and vehicle. DMSO, dimethyl sulfoxide; IVF, *in vitro* fertilization; LP, LP12 hydrochloride hydrate.
